# The Heparan Sulfate Proteoglycan Glypican-6 Is Upregulated in the Failing Heart, and Regulates Cardiomyocyte Growth through ERK1/2 Signaling

**DOI:** 10.1371/journal.pone.0165079

**Published:** 2016-10-21

**Authors:** Arne O. Melleby, Mari E. Strand, Andreas Romaine, Kate M. Herum, Biljana Skrbic, Christen P. Dahl, Ivar Sjaastad, Arnt E. Fiane, Jorge Filmus, Geir Christensen, Ida G. Lunde

**Affiliations:** 1 Institute for Experimental Medical Research, Oslo University Hospital and University of Oslo, Oslo, Norway; 2 Center for Heart Failure Research, University of Oslo, Oslo, Norway; 3 Department of Cardiothoracic Surgery, Oslo University Hospital, Oslo, Norway; 4 Research Institute of Internal Medicine, Oslo University Hospital, Oslo, Norway; 5 Division of Molecular and Cellular Biology, Sunnybrook Research Institute and Department of Medical Biophysics, University of Toronto, Toronto, Canada; University of Patras, GREECE

## Abstract

Pressure overload is a frequent cause of heart failure. Heart failure affects millions of patients worldwide and is a major cause of morbidity and mortality. Cell surface proteoglycans are emerging as molecular players in cardiac remodeling, and increased knowledge about their regulation and function is needed for improved understanding of cardiac pathogenesis. Here we investigated glypicans (GPC1-6), a family of evolutionary conserved heparan sulfate proteoglycans anchored to the extracellular leaflet of the cell membrane, in experimental and clinical heart failure, and explored the function of glypican-6 in cardiac cells *in vitro*. In mice subjected to pressure overload by aortic banding (AB), we observed elevated glypican-6 levels during hypertrophic remodeling and dilated, end-stage heart failure. Consistently, glypican-6 mRNA was elevated in left ventricular myocardium from explanted hearts of patients with end-stage, dilated heart failure with reduced ejection fraction. Glypican-6 levels correlated negatively with left ventricular ejection fraction in patients, and positively with lung weight after AB in mice. Glypican-6 mRNA was expressed in both cardiac fibroblasts and cardiomyocytes, and the corresponding protein displayed different sizes in the two cell types due to tissue-specific glycanation. Importantly, adenoviral overexpression of glypican-6 in cultured cardiomyocytes increased protein synthesis and induced mRNA levels of the pro-hypertrophic signature gene ACTA1 and the hypertrophy and heart failure signature genes encoding natriuretic peptides, NPPA and NPPB. Overexpression of GPC6 induced ERK1/2 phosphorylation, and co-treatment with the ERK inhibitor U0126 attenuated the GPC6-induced increase in NPPA, NPPB and protein synthesis. In conclusion, our data suggests that glypican-6 plays a role in clinical and experimental heart failure progression by regulating cardiomyocyte growth through ERK signaling.

## Introduction

Heart failure is a syndrome affecting millions of people worldwide and is currently one of the main causes of morbidity and mortality, carrying huge monetary costs for society [1]. Pressure overload, as in patients with hypertension or aortic stenosis (AS), is a common stimulus for cardiac remodeling and eventually cardiac dysfunction and failure [2]. Cardiac remodeling encompasses cellular and extracellular matrix alterations in the heart, including cardiomyocyte hypertrophy and apoptosis, and fibrosis [3]. To provide new and effective therapies for heart failure, an improved understanding of underlying molecular mechanisms is necessary [4].

We and others have established that proteoglycans, proteins substituted with glycosaminoglycans (GAG) chains, play important roles during cardiac remodeling and failure. For instance, syndecan-4 regulates aspects of fibrosis, inflammation and cardiomyocyte hypertrophy in response to pressure overload [5–9]. Syndecans (SDC1-4) and glypicans (GPC1-6) are cell surface proteoglycans and the main sources of cell surface heparan sulfate (HS) GAGs [10, 11]. In contrast to the transmembrane syndecans, glypicans are tethered to the extracellular leaflet of the plasma membrane by a glycosylphosphatidylinositol (GPI) anchor. Although glypicans are expressed in the heart [12, 13], their roles in cardiac remodeling and failure are unknown.

Glypicans regulate cellular responses to various growth factors [14]. These growth factors are known to regulate cell migration, differentiation and proliferation [15], emphasizing the diversity of cellular processes potentially affected by glypicans. For instance, bone morphogenetic proteins (BMPs) are interaction partners of glypicans [16–19]. Despite being named according to their effects on bone and cartilage formation [20], BMPs exert effects in multiple tissues [21, 22]. Like other members of the transforming growth factor (TGF) family, BMPs signal through Smad and mitogen-associated protein kinases (MAPKs) such as ERK1/2 [23]. BMP4 stimulates hypertrophy, apoptosis and fibrosis in the heart [24], suggesting a link between cardiac remodeling, BMPs and glypicans. Furthermore, mutations in the GPC3 and GPC6 genes are found in patients with rare, familial growth disorders, e.g. the Simpson-Golabi-Behmel overgrowth syndrome and the generalized omodysplasia (OMOD1) dwarfism syndrome, respectively [25–29]. Congenital heart defects have been reported in both syndromes [30, 31], indicating important roles of GPC6 and GPC3 in the heart.

In the present study, we explored the regulation of glypican expression in cardiac tissue and cells in clinical and experimental heart failure, and investigated interactions between glypicans, BMP4, MAPK signaling, pro-fibrotic and pro-hypertrophic growth processes in cardiac cells.

## Methods

### Ethics

Human studies were reviewed and approved by the Regional Committee for Medical Research Ethics (REC), permit of approval number 07482a, the South-Eastern Regional Health Authority, Norway, and conformed to the Declaration of Helsinki. Written informed consent was obtained from all patients and from next of kin for controls. Animal experiments were reviewed and approved by the Norwegian National Animal Research Committee (permit of approval number 3170) and conformed to the Guide for the Care and Use of Laboratory Animals (NIH publication No. 85–23, revised 2011, US).

### Mouse Heart Failure Model

Chronic pressure overload was induced in eight week old wild-type (WT) (C57BL/6JBomTac, Taconic, Skensved, Denmark) mice by banding of the ascending aorta (AB), as previously described [5, 32]. Sham-operated animals were subjected to the same procedure, without tightening of the suture around the aorta. AB or sham surgery was performed on intubated and ventilated mice breathing a mixture of 98% oxygen and 2% isoflurane. Subcutaneous injection of 0.02 ml buprenorphine (0.3 mg/ml) was administered post-operatively. Echocardiographic examinations were performed by an experienced researcher blinded to type of surgery, on mildly anaesthetized mice breathing 1.75% isoflurane on a mask, using the VEVO 2100 system (VisualSonics, Toronto, Canada). Animals with sufficient degree of aortic constriction (maximal flow velocity (Vmax) ≥ 4 m/s over the stenosis) were included. Mice were sacrificed by cervical dislocation under deep anesthesia 1, 3, 16 or 18 weeks post surgery. Hearts and lungs were rapidly excised, the left ventricle (LV) dissected, rinsed in 1X phosphate-buffered saline (PBS), snap-frozen in liquid nitrogen and stored at -70°C. Body weight, LV weight (LVW) and lung weight (LW) were recorded at sacrifice, and LVW and LW normalized to tibia length (TL).

### Myocardial Tissue Samples from Heart Failure Patients

LV tissue samples were obtained from beating hearts immediately after explantation from patients with end-stage, dilated heart failure with reduced ejection fraction (HFrEF) undergoing cardiac transplantation. LV samples from non-diseased hearts considered, but found unsuitable for transplantation, were sampled as controls. Patients were characterized according to hospital guidelines. Tissue samples were snap-frozen in liquid nitrogen and stored at -70°C.

### Primary Cultures of Cardiac Fibroblasts and Myocytes

Primary ventricular cells were isolated from Wistar rats 1–3 days of age, i.e. neonatal cardiac fibroblasts (NFB) and myocytes (NCM), as described [8]. Briefly, hearts were excised and trimmed for atrial tissue prior to mechanical digestion in a collagenase/pancreatin solution.

Primary ventricular cells were isolated from WT mice, i.e. adult fibroblasts (AFB), as described [5, 6]. Hearts were cannulated through the aorta and perfused retrogradely with collagenase solution (pH = 7.6, 25mM Hepes, 130mM NaCl, 5.4mM KCl, 0.4mM NaH_2_PO_4_, 0.5mM MgCl_2_, 22mM glucose monohydrate and 424 u/ml collagenase II (Worthington, Lakewood, NJ)) for 10 min before the LV was excised and shredded in the collagenase flow-through. The neonatal and adult ventricular cells were transferred to uncoated culture flasks with serum-containing medium for 20 min for fibroblast attachment. The unattached NCM fraction was seeded onto gelatine/fibronectin-coated six-well plates in serum-containing medium at a density of 3.75 x 10^5^ cells/ml. NFB and AFB were cultured in serum-containing medium for up to one week, passaged and seeded onto six-well plates at a density of 1.8 x 10^5^ cells/ml. Cells were cultured in a 37°C, 5% CO_2_ humidified incubator. The purity of similar NFB and NCM cultures has been confirmed by an 800-fold higher expression of cardiac troponin I (TNNI) in NCM vs. NFB [8].

Viral transduction of cells cultured in serum-containing medium was performed using adenovirus serotype 5 vectors encoding human GPC6 (AdhGPC6, Vector Biolabs, Malvern, PA) or empty vector control (AdNull, Vector Biolabs) for 24 h. Virus titers were 2 x 10^6^ plaque forming units (PFU)/ml medium for NFB and 3.75 x 10^6^ PFU/ml for NCM. Prior to treatment, cells were serum-starved for 24 h. Cells were treated for 24 h with serum-free medium with or without tumor necrosis factor (TNF)α (50ng/ml, PMC3014, Gibco, Gaithersburg, MD), interleukin (IL)-1β (10ng/ml, PRC0814, Gibco), transforming growth factor (TGF)β1 (10ng/ml, GF111, Merck Millipore, Darmstadt, Germany), IL-18 (100ng/ml, 521-RL, R&D Systems, Minneapolis, MN), norepinephrine (NE; 100μM, A7257, Sigma-Aldrich, St. Louis, MO), basic fibroblast growth factor (FGF2; 20ng/ml, 450–33, PeproTech, Rocky Hill, NJ), platelet-derived growth factor (PDGF)-BB (20ng/ml, 220-BB-010, R&D Systems), angiotensin (ANG)II (1μM, A9525, Sigma-Aldrich) or BMP4 (50ng/ml, 314-BP, R&D Systems), before harvest of RNA or protein. Non-treated cells were used as controls. Experiments were conducted in three separate cell culture isolations.

### HEK293 and NIH 3T3 Cell Cultures

Human endothelial kidney (HEK)293 cells were transfected with a pcDNA3.1 plasmid encoding human GPC6 [33] using Lipofectamine 2000 (Invitrogen, Paisley, UK), as described [5]. Cells transfected with empty pcDNA3.1 (vehicle) served as control. BMP4 (50ng/ml, R&D Systems) was added 24 h after transfection and protein harvested after 180 min. Non-stimulated cells were used as controls. NIH 3T3 fibroblasts (CRL-1658, ATCC, Manassas, VA) were treated with BMP4 (50ng/ml, R&D Systems) for 24 h prior to harvest of cell RNA or protein. HEK293 and NIH 3T3 cells were cultured according to supplier protocols, essentially as described [5, 8].

### Cardiac Fibroblast Migration Assay

The NFB migration scratch assay was performed essentially as described [6]. NFB cells seeded in six-well plates at a density of 1 x 10^5^ cells/ml were transduced with AdNull or AdhGPC6 for 24 h prior to serum starvation for 24 h. A 0.7 mm wide vertical scratch was made with a cell scraper across each well where four horizontal lines 0.5 cm apart had been marked under the culture dish. Serum free medium was added with or without BMP4 (50ng/ml, R&D Systems). Images of 1–3 areas of n = 3–6 wells per condition were taken at 0, 12, 24, 48, 72 and 96 h following scratch using an Eclipse Ts100 phase contrast microscope (Nikon, Tokyo, Japan) and the cell free area was measured in ImageJ (NIH) and normalized to the area measured at the time of scratch (time 0) to quantify migration (%).

### Cardiomyocyte Radioactive [^3^H] Leucine Incorporation Protein Synthesis Assay

The radioactive [^3^H] leucine incorporation protein synthesis assay was performed and quantified essentially as described [34]. In brief, NCM were transduced with AdNull or AdhGPC6 for 24 h before being cultured in serum-free medium containing 1.25 μCi/ml [^3^H] leucine (American Radiolabel Chemicals, St Louis, MO) for 48 h, with or without 10μM of the dual specificity kinase (MEK1/2) inhibitor U0126 (Promega, Madison, WI). At harvest, cells were washed in 95% ethanol and lysed in 0.2M NaOH. Lysates were diluted in Pico-Fluor 40 (PerkinElmer) and [^3^H] leucine incorporation quantified as counts per min (CPM) using the Wallac Winspectral 1414 liquid scintillation counter (PerkinElmer). Samples were measured in duplicates and serum-cultured NCM were used as positive control.

### RNA Isolation and Quantitative Real-Time PCR (qRT-PCR)

RNA was isolated from cells and LV tissue using RNeasy mini (74106, Qiagen Nordic, Oslo, Norway), as described [8]. RNA concentration was measured using the Nanodrop ND-1000 Spectrophotometer (Thermo Fisher Scientific, Waltham, MA). Samples with RNA integrity number (RIN) >7 determined on the 2100 Bioanalyzer (Agilent Technologies, Santa Clara, CA) were accepted. cDNA synthesis was performed using iScript cDNA Synthesis Kit (Bio-Rad Laboratories, Inc., Hercules, CA) according to manufacturer’s protocol. Gene expression levels were measured by qRT-PCR using TaqMan assays (Applied Biosystems, Foster City, CA) on an ABI PRISM 7900HT Sequence Detection System (Applied Biosystems). TaqMan assays used were: mouse GPC1 (Mm00497305_m1), mouse GPC2 (Mm00549650_m1), mouse GPC3 (Mm00516722_m1), mouse GPC4 (Mm00515035_m1), mouse GPC5 (Mm00615599_m1), mouse GPC6 (Mm00516235_m1), rat GPC6 (Rn01466046_m1), human GPC6 (Hs00170677_m1), mouse BMP4 (Mm00432087_m1), human BMP4 (Hs03676628_s1), mouse COL1A2 (Mm00483888_m1), rat COL1A2 (Rn01526721_m1), mouse COL3A1 (Mm01254476_m1), rat COL3A1 (Rn01437681_m1), mouse LOX (Mm00495386_m1), rat LOX1 (Rn01491829_m1), mouse ACTA2 (Mm00725412_s1), rat ACTA2 (Rn01759928_g1), mouse NPPA (Mm01255747_g1), rat NPPA (Rn00664637_g1), mouse NPPB (Mm01255770_g1), rat NPPB (Rn00580641_m1), mouse ACTA1 (Mm00808218_g1), mouse ACTA1 (Mm00808218_g1), rat ACTA1 (Rn01426628_g1), rat PCNA (Rn01514538_g1), mouse B2M (Mm00437762_m1), rat RPL4 (Rn00821091_g1), mouse RPL32 (Mm02528467_g1) and human RPL32 (custom made for Rpl32 exon 32 [8]). Results were analyzed using the Sequence Detection System 2.3 software (Applied Biosystems).

### Protein Isolation and Immunoblotting

Mouse and human LV tissues were homogenized using a Polytron 1200 in a 1X PBS-based lysis buffer containing 1% Triton X-100 (Sigma, MI), 0.1% Tween-20 (Sigma), 0.1% sodium dodecyl sulfate (SDS), protease inhibitors (Complete EDTA-free tablets, Roche Diagnostics, Oslo, Norway) and phosphatase inhibitors (PhosStop, Roche Diagnostics), as described [5, 8]. Whole cell lysates from primary heart, HEK293 and NIH 3T3 cultures were harvested using the same lysis buffer, as described [8]. All samples were spun at 20 000 g for 10 min at 4°C and the supernatant stored at -70°C. Protein concentrations were measured using Micro BCA kit (Thermo Fisher Scientific, Waltham, MA). Protein lysates used for assessment of heparan sulfate proteoglycans were methanol (MetOH) precipitated prior to incubation with heparan sulfate degrading enzymes (Heparitinase I, Heparitinase II, Heparitinase III and Chondroitinase cABC (all from AMSBIO, Abingdon, UK)), as described [8, 35, 36]. SDS-PAGE separation was performed on Criterion, 4–15% Tris-HCL gels under reducing and non-reducing conditions using sample buffer with or without dithiothreitol (DTT), respectively, and proteins transferred to PVDF membranes using the Trans-Blot Turbo Transfer System according to protocol (Biorad). Membranes were blocked in non-fat dry milk (Sigma), casein (Roche Diagnostics, Oslo, Norway) or BSA (Bio-Rad) prior to incubation with primary and secondary antibodies. Primary antibodies used were: anti-glypican-6 (AF2845, R&D)[33], anti-Δ3G10 (#3708660–1, Amsbio, Abington, UK), anti-β2 microglobulin (B2M; ab75853, Abcam, Cambridge, UK), anti-phospho Thr200/Tyr204 ERK1/2 (#9101, Cell Signaling Technology, Danvers, MA), anti-ERK1/2 (#9102, Cell Signaling Technology), anti-vinculin (V9131, Sigma-Aldrich) and anti-GAPDH (sc-20357, Santa Cruz Biotechnology, Dallas, TX). Recombinant human His-tagged GPC6 produced in *E*.*coli* (80R-3484, Fitzgerald Industries, Acton, MA) was used as positive control. Membranes were developed using ECL Prime (Amersham/GE HealthCare, Buckinghamshire, UK) in the Las-4000 (Fujifilm, Tokyo, Japan), followed by stripping with Western blot stripping buffer (210591, Thermo Scientific) and reprobing. Images were quantified and processed using ImageJ (NIH) and Photoshop CS5.

### Statistics

Data are given as group means ± S.E.M. All statistical tests were performed in GraphPad Prism 6.01 with statistical significance accepted at p<0.05. The statistical tests applied were unpaired Student’s *t*-test, one-way ANOVA with Bonferroni post-hoc test, and Pearson correlation.

## Results

### Glypican-6 Is Upregulated in the Failing Mouse Heart

**Fig 1:**
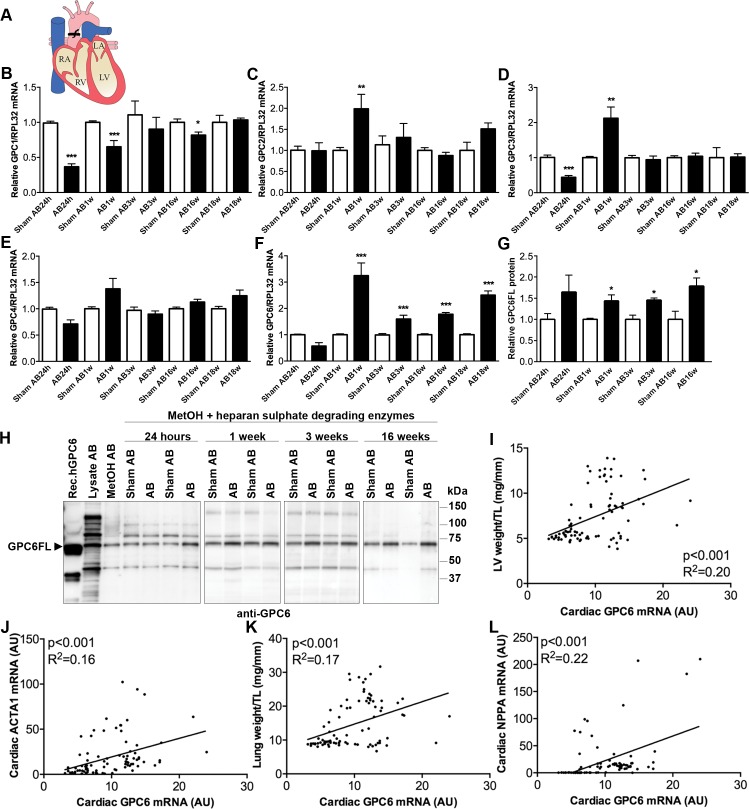
Glypican-6 expression is increased in the failing mouse heart. Schematic of the aortic banding (AB) heart failure model with heart regions indicated (A). Chronic pressure overload was induced in adult mice by banding of the ascending aorta. RA: right atrium, RV: right ventricle, LA: left atrium, LV: left ventricle. Relative LV mRNA levels of GPC1 (B), GPC2 (C), GPC3 (D), GPC4 (E) and GPC6 (F) after 24h, 1, 3, 16 and 18 weeks of AB or sham-operation in male mice (n = 3–10). See Table 1 for animal characteristics. mRNA expression was normalized to ribosomal protein L32 (RPL32) expression. Representative immunoblots and quantitative data of full length GPC6 (GPC6FL; Mw ≈62kDa) in LV protein lysates from AB- and sham-operated control mice analyzed under reducing conditions (+ dithiothreitol). For immunoblotting of heparan sulfate (HS) proteoglycans in tissue, proteoglycans were methanol (MetOH) precipitated prior to digestion with heparan sulfate degrading enzymes [8, 36](G and H; n = 3 at all time-points). Recombinant human GPC6 produced in *E*.*coli* was used as positive control (Rec.hGPC6). Data are presented as mean ± S.E.M. Unpaired Student’s *t-test* was used for statistical testing vs. controls at respective time-points. *P<0.05; **P<0.01; ***P<0.001. Pearson correlations of LV GPC6/RPL32 mRNA vs. LV weight/tibia length (TL)(I), ACTA1/RPL32 mRNA (J), lung weight/TL (K) and NPPA/RPL32 mRNA (L) in AB- and sham-operated mice (24h-18 weeks).

To investigate whether glypican expression was regulated in the failing heart, we measured cardiac GPC1-6 transcript levels in mice subjected to pressure overload induced by AB (Fig 1A and Table 1). We examined the cardiac phenotype after AB by echocardiography, and harvested LV during the acute phase (i.e. 24 h post-AB), during hypertrophic remodeling (1 and 3 weeks post-AB) and during end-stage, dilated heart failure (16 and 18 weeks post-AB).

**Table 1:** Characterization of mice subjected to aortic banding.

	Sham AB24h	AB24h	ShamAB1w	AB1w	ShamAB3w	AB3w	ShamAB16w	AB16w	Sham AB18w	AB18w
*Animal and organ weights*										
N	10	10	10	9	10	9	10	9	5	8
Body weight (g)	25.0 ± 0.9	23.5 ± 0.4	26.8 ± 0.4	26.3 ± 0.5	26.4 ± 0.5	24.1 ± 0.6*	34.4 ± 1.2	23.4 ± 1.2***	36.4 ± 2.4	25.9 ± 1.9**
LVW/TL (mg/mm)	4.82 ± 0.17	5.84 ± 0.44*	5.09 ± 0.06	8.25 ± 0.21***	5.24 ± 0.18	8.87 ± 0.19***	5.85 ± 0.10	12.41 ± 0.33***	5.93 ± 0.38	12.22 ± 0.16***
LW/TL (mg/mm)	8.51 ± 0.26	13.16 ± 1.07***	8.95 ± 0.20	20.01 ± 1.40***	8.99 ± 0.15	20.40 ± 1.53***	9.38 ± 0.11	23.87 ± 1.29***	9.61 ± 0.34	24.51 ± 1.51***
*M-mode echocardiography*										
N	0	0	6	7	8	6	6	9	3	8
LAD (mm)	NA	NA	1.70 ± 0.02	3.02 ± 0.09***	1.81 ± 0.02	3.20 ± 0.13***	1.70 ± 0.09	3.49 ± 0.20***	1.80 ± 0.06	3.63 ± 0.19***
LVPWd (mm)	NA	NA	0.75 ± 0.03	1.08 ± 0.02***	0.75 ± 0.03	1.12 ± 0.02***	0.82 ± 0.05	1.08 ± 0.02***	0.82 ± 0.01	1.06 ± 0.04**
LVIDd (mm)	NA	NA	4.08 ± 0.11	4.09 ± 0.05	4.27 ± 0.09	4.05 ± 0.12*	4.02 ± 0.19	5.87 ± 0.12***	4.26 ± 0.05	5.75 ± 0.14***
FS (%)	NA	NA	18.87± 1.29	14.29 ± 1.22*	20.94 ± 0.91	15.20 ± 1.68*	18.09 ± 1.66	6.81 ± 0.84***	15.25 ± 1.66	6.65 ± 0.89***
*Left ventricular mRNA expression*										
N	10	10	10	9	10	9	10	9	3	8
NPPA/ RPL32	1.0 ± 0.5	3.2 ± 0.7*	1.0 ± 0.1	11.0 ± 1.65***	1.0 ± 0.5	9.8 ± 0.7***	1.0 ± 0.1	48.4 ± 4.9***	1.0 ± 0.2	78.7 ± 16.3*
NPPB/ RPL32	1.0 ± 0.1	11.5 ± 1.5***	1.0 ± 0.1	3.0 ± 0.4***	1.0 ± 0.1	2.3 ± 0.2***	1.0 ± 0.1	6.2 ± 0.2***	1.0 ± 0.5	8.5 ± 0.4***
ACTA1/ RPL32	1.0 ± 0.2	1.7 ± 0.4*	1.0 ± 0.2	6.5 ± 1.3***	1.0 ± 0.2	7.0 ± 0.9***	1.0 ± 0.2	4.6 ± 1.3**	1.0 ± 0.5	11.4 ± 2.2*

Post-mortem organ weights, M-mode echocardiographic recordings and left ventricular mRNA expression data of male mice 24h, 1, 3, 16 and 18 weeks after aortic banding (AB), and of sham-operated controls for each time-point. Statistical differences were tested using an unpaired t-test vs. respective sham-operated controls

*p<0.05

**p<0.01

***p<0.001. Relative mRNA expression of the hypertrophic marker gene skeletal muscle α-actin (ACTA1), heart failure signature molecules atrial (NPPA/ANP) and brain (NPPB/BNP) natriuretic peptides normalized to ribosomal protein L32 (RPL32) expression. LVW, left ventricular weight; TL, tibia length; LW, lung weight; LAD, left atrial diameter; LVPWd, left ventricular posterior wall thickness in diastole; LVIDd, left ventricular internal diameter in diastole; FS, fractional shortening; NA, not available.

GPC1-3 mRNA levels showed minor alterations during the acute phase and during hypertrophic remodeling, and returned to levels of sham-operated controls during end-stage, dilated heart failure (Fig 1B–1D, respectively). Cardiac GPC4 mRNA was unaltered in response to AB (Fig 1E). We were unable to detect GPC5 mRNA in the LV of mice. Interestingly, cardiac GPC6 mRNA was upregulated during hypertrophic remodeling (2.7-fold at AB1w and 1.7-fold at AB3w vs. respective controls) and end-stage dilated heart failure (1.8-fold at AB16w and 2.2-fold at AB18w; Fig 1F).

To quantify glypican-6 protein in heart tissue, proteoglycans in total protein lysates were precipitated with methanol prior to heparan sulfate digestion. The anti-heparan sulfate Δ3G10 antibody recognizes the heparan sulfate neo-epitope after digestion, confirming successful digestion (S1 Fig). Importantly, and in line with increased GPC6 mRNA, levels of full-length GPC6 (GPC6FL; Mw≈ 62 kDa) were upregulated during hypertrophic remodeling (1.4-fold at AB1w and 1.5-fold at AB3w) and during end-stage dilated heart failure (1.8-fold at AB16w; Fig 1G and 1H).

To investigate whether increased GPC6 levels were associated with severity and progression of disease, GPC6 mRNA in sham- and AB-operated mouse hearts was correlated with LV weight, expression of the hypertrophic signature gene ACTA1 (encoding α-skeletal actin), lung weight and heart failure signature genes NPPA and NPPB (encoding atrial and brain natriuretic peptides, ANP and BNP, respectively). As expected, ACTA1, NPPA and NPPB were increased after AB, being elevated at all time-points after AB (Table 1). ACTA1 was elevated 1.7-fold at AB24h, with levels 4.6–11.4-fold higher than sham controls at AB1w-18w. NPPB reached a maximum increase at AB24h with levels 11.5-fold higher than sham controls, and showed levels 2.3–8.5-fold higher than controls at AB1w-18w. NPPA, unlike NPPB, showed a 3.2-fold increase at AB24h, with levels 9.8–11.0-fold at AB1w-3w and reaching a maximum of 48.4–78.7-fold increase at AB16-18w. LV GPC6 expression levels correlated positively with degree of LV remodeling (vs. LV weight R^2^ = 0.20, vs. ACTA1 expression R^2^ = 0.16; Fig 1I and 1J) and congestive heart failure (vs. lung weight R^2^ = 0.17, vs. NPPA expression R^2^ = 0.22; Fig 1K and 1L), suggesting that increased GPC6 levels were associated with increased disease severity. As expected from the differences in expression kinetics of NPPB and GPC6 after AB, there was no correlation between these two transcripts (data not shown). Altogether, the upregulation of GPC6 mRNA and protein during hypertrophic remodeling and dilatation in response to LV pressure overload suggests a role for GPC6 in heart failure progression.

### Glypican-6 Is Upregulated in the Failing Human Heart

**Fig 2:**
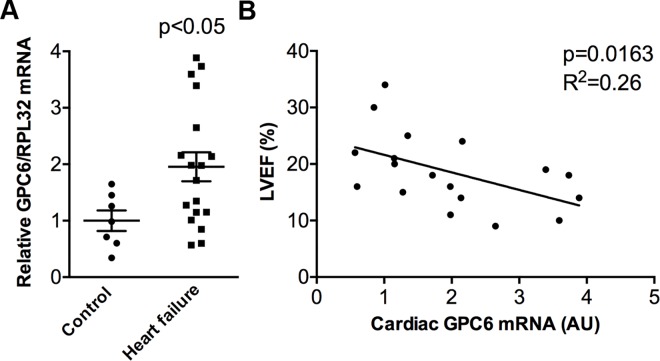
Glypican-6 expression is upregulated in the failing human heart. Relative GPC6 mRNA levels in left ventricular biopsies from end-stage heart failure patients (n = 18) compared to controls (n = 7) (A). See Table 2 for patient characteristics. GPC6 mRNA levels were normalized to ribosomal protein L32 (RPL32) expression and data presented as individual data points. Unpaired Student’s *t-test* was used to test for statistical significance. Pearson correlation between left ventricular GPC6 mRNA level and ejection fraction (EF) in end-stage heart failure patients (n = 18) (B).

We next assessed GPC6 expression in failing human hearts. GPC6 mRNA levels were measured in LV samples from explanted hearts of patients with end-stage, dilated heart failure with reduced ejection fraction (HFrEF) undergoing cardiac transplantation. The severely impaired pumping function was evident by EF of 19.17±1.58%, and dilatation was evident by internal diameter in diastole (LVIDd) of 7.41±0.06 cm (Table 2). GPC6 mRNA was 1.95-fold higher in patients than in controls (Fig 2A). Importantly, GPC6 expression correlated negatively with EF (Fig 2B), suggesting, like in mice, that increased GPC6 levels were associated with more progressed heart failure.

**Table 2:** Characterization of patients with end-stage heart failure.

	Heart failure patients (n = 18)
Age (years)	48.9 ± 2.4
Gender	n = 14 males, n = 4 females
NYHA class	n = 8 NYHA III, n = 10 NYHA IV
Pro-BNP (pmol/L)	417.0 ± 85.0
BMI (kg/m^2^)	26.0 ± 0.7
LVEF (%)	19.2 ± 1.6
LVEDV (ml)	279.0 ± 23.2
LVESV (ml)	227.9 ± 21.7
SV (ml)	51.1 ± 3.6
WMSI	2.42 ± 0.04
IVSd (cm)	0.81 ± 0.05
LVPWd (cm)	0.71 ± 0.02
LVIDd (cm)	7.41 ± 0.22

Clinical and echocardiography data (mean±SEM) of patients with end-stage heart failure with reduced ejection fraction (HFrEF). Non-diseased hearts considered for transplantation but deemed unsuitable were used as control samples (age 46.9 ± 7.5 years, n = 4 males and n = 3 females). NYHA, New York Heart Association; BMI, body mass index; BNP, brain natriuretic peptide; LVEF, left ventricular ejection fraction; WMSI, wall motion score index [8]; LVEDV, LV end diastolic volume; LVESV, LV end systolic volume; SV, stroke volume; IVSd, interventricular septal diameter in diastole; LVIDd, LV internal diameter in diastole; LVPWd, LV posterior wall thickness in diastole.

### Cardiac Fibroblasts and Cardiomyocytes Produce Glypican-6

**Fig 3:**
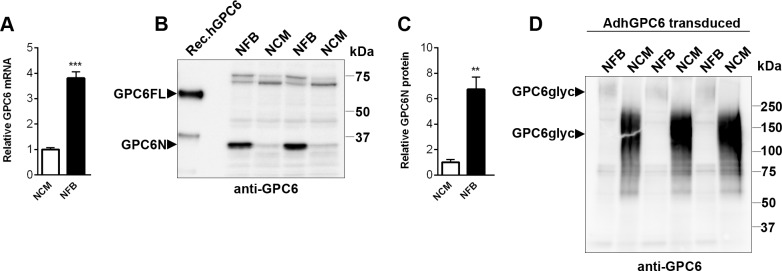
Glypican-6 is mainly produced by cardiac fibroblasts in the heart. Relative GPC6 mRNA in rat heart neonatal fibroblasts (NFB) and cardiomyocytes (NCM; A), n = 13–15 from three separate cell cultures. Immunoblot and quantification of the N-terminal GPC6 (GPC6N; Mw ≈35kDa) from NFB and NCM cell lysates analyzed under reducing conditions (+ dithiothreitol; B and C), n = 3. Recombinant human GPC6 produced in *E*.*coli* was used as positive control (Rec.hGPC6). Immunoblot of glycanated GPC6 (GPC6glyc; Mw>150kDa) in protein lysates from NFB and NCM transduced with an adenovirus encoding human GPC6 (AdhGPC6) run under non-reducing conditions (-dithiothreitol; D). Data are presented as mean ± S.E.M. Unpaired Student’s *t-*test (A and C) was used to test for statistical significance. **P<0.01; ***P<0.001; significantly different from other cell type.

GPC6 levels were examined in cultured cardiac fibroblasts and myocytes. GPC6 mRNA expression was found in both cell types, and was 3.8-fold higher in NFB compared to NCM (Fig 3A).

Due to post-translational cleavage, glypican protein chemistry typically reveals several GPC6 protein bands by immunoblotting [33, 37]. Cleavage results in an N-terminal domain and a C-terminal HS GAG substituted domain linked together by disulfide bridges [33]. Protein lysate preparation under reducing conditions (e.g. +DTT in sample buffer) separates the two subunits, while non-reducing conditions (e.g. -DTT) keep the subunits together. In order to quantify GPC6 protein in heart cells, we characterized GPC6 immunoblotting bands in cardiac fibroblasts and myocytes with and without overexpression of GPC6 by viral transduction (S2A–S2C Fig), using a polyclonal GPC6 antibody recognizing epitopes in the N-terminal domain [33].

Immunoblot analysis of NFB (S2A Fig) and NCM (S2B Fig) samples under non-reducing conditions revealed the expected proteoglycan smear in the high kDa range corresponding to the HS-substituted full length GPC6 (GPC6glyc >150 kDa), along with the ≈35 kDa N-terminal GPC6 domain (GPC6N). Analyzing the same samples under reducing conditions revealed the expected reduction in GPC6glyc and increase in GPC6N. The non-glycanated, uncleaved full-length core protein (GPC6FL; 555 amino acids) was found in both sample conditions with the expected size, i.e. ≈62 kDa. Recombinant human GPC6 (Rec.hGPC6) run under reducing conditions revealed bands similar to that of cardiac cells, i.e. GPC6FL and GPC6N. Precipitation of proteoglycans with methanol and digesting HS with heparitinase prior to analysis under non-reducing conditions, yielded the expected GPC6FL band at ≈62 kDa and lower GPCN level compared to reducing conditions (S2C Fig). As these *in vitro* data suggested that GPC6N under reducing conditions (+DTT in sample buffer) was representative for full-length GPC6 protein levels, we used GPC6N levels as a measure of GPC6 abundance in cells throughout the study. The schematic cartoons in S2D Fig illustrate the GPC6 protein chemistry.

Immunoblotting showed that GPC6N protein was produced in both cell types, and was 6.5-fold higher in NFB than in NCM (Fig 3B and 3C), consistent with the higher mRNA expression in NFB. Adenoviral overexpression of GPC6 yielded distinct GPC6glyc bands in NFB and NCM, as seen under non-reducing conditions (Fig 3D), suggesting differential GPC6 glycanation in the two cardiac cell types. Whereas GPC6glyc in NFB was >250kDa, NCM mainly produced GPC6 with shorter HS GAG chains, i.e. >150kDa.

### Cardiac Fibroblast Glypican-6 Expression Is Regulated by ANGII and BMP4

To identify direct regulators of cardiac GPC6 expression, we treated cultured fibroblasts with inflammatory mediators, growth and neurohumoral factors central to heart failure. ANGII slightly upregulated GPC6 mRNA expression in NFB (1.4-fold; S3A Fig). In contrast, IL-1β, TGFβ1, FGF2, PDGF-BB and NE down-regulated NFB GPC6 expression (S3A Fig). TNFα and IL-18 had no effect on NFB GPC6 mRNA levels (S3A Fig).

**Fig 4:**
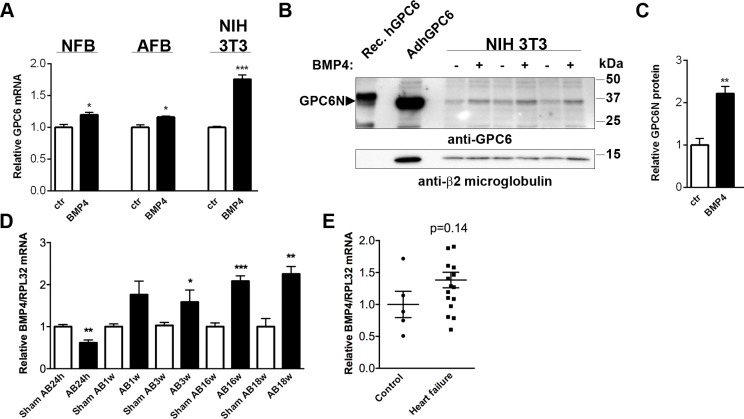
Glypican-6 expression is regulated by BMP4 in cardiac fibroblasts. Relative GPC6 mRNA in neonatal rat cardiac fibroblasts (NFB), adult mouse cardiac fibroblasts (AFB) and NIH 3T3 fibroblasts treated with bone morphogenetic protein (BMP)4, compared to non-stimulated controls (A), n = 3–26. Immunoblot and quantification of the N-terminal GPC6 (GPC6N; Mw ≈35kDa) analyzed under reducing conditions (+dithiothreitol) in NIH 3T3 cells stimulated with BMP4 for 24 h compared to non-stimulated controls (B and C), n = 3. Recombinant human GPC6 (Rec.hGPC6) and protein lysate from NFB overexpressing GPC6 (AdhGPC6) were used as positive controls and β-2-microglobulin was used for loading control. Relative left ventricular BMP4 mRNA after 24h, 1, 3, 16 and 18 weeks of aorta banding (AB) or sham-operation in male mice (D; n = 3–10, see Table 1 for animal characteristics). BMP4 mRNA in left ventricular biopsies from end-stage heart failure patients (n = 17), compared to controls (n = 5) (E). BMP4 mRNA was normalized to ribosomal protein L32 (RPL32) in D and E. Data are presented as mean ± S.E.M. Unpaired Student’s *t-test* were used to test for statistical significance. *P<0.05; **P<0.01; ***P<0.001; experimental/disease group different from control.

Given the suggested interaction between glypicans and BMPs in previous studies [16–19], we treated NFB, AFB and NIH 3T3 fibroblasts with BMP4, a BMP isoform important in heart failure [24]. Interestingly, BMP4 increased GPC6 mRNA in all three fibroblast types tested (1.2-fold, 1.2-fold and 1.5-fold, respectively; Fig 4A). The increase in GPC6 expression was confirmed at protein level, with GPC6N level being 2.2-fold higher after BMP4 treatment in NIH 3T3 (Fig 4B and 4C). To investigate whether increased BMP4 levels were associated with increased GPC6 expression after AB in mice and human heart failure, we measured BMP4 mRNA in the LV. BMP4 mRNA was increased during hypertrophic remodeling (1.81-fold at AB3w) and during end-stage dilated heart failure (2.15-fold at AB16w and 2.07-fold at AB18w; Fig 4D), i.e. similar expression pattern to GPC6 (Fig 1F). These results supported that increased BMP4 could constitute a signal responsible for the upregulation of GPC6 *in vivo*. In patients, there was a tendency for increased BMP4 mRNA (1.38-fold, p = 0.14; Fig 4E).

Finally, of the inflammatory mediators, growth and neurohumoral factors that we tested none upregulated GPC6 mRNA in NCM. BMP4, NE, TNFα and IL-1β down-regulated GPC6 mRNA, whereas ANGII, TGFβ1 and IL-18 had no effect (S3B Fig).

### Glypican-6 Enhances BMP4-Dependent ERK1/2 Signaling in Cultured Cardiac Fibroblasts

**Fig 5:**
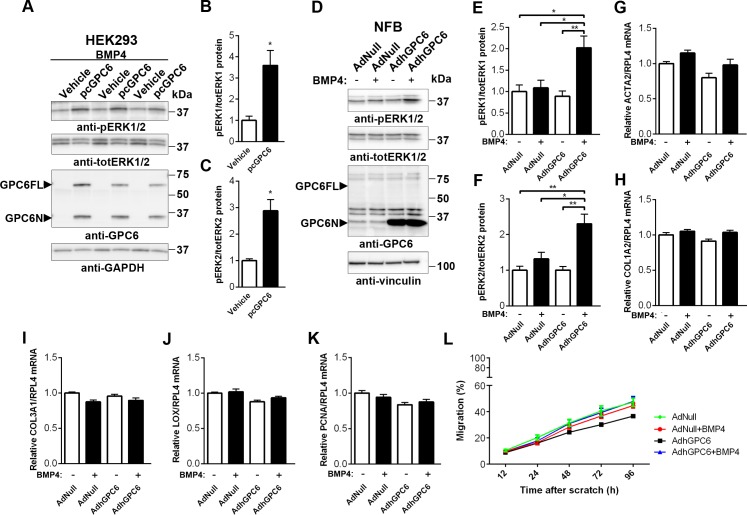
Glypican-6 enhances BMP4-dependent ERK1/2 signaling in cultured cardiac fibroblasts. Representative immunoblots and quantification of phospho-extracellular signal-regulated kinase (ERK)1 (pERK 44) relative to total ERK1 (totERK 44; Mw ≈44 kDa) and phospho-ERK2 (pERK 42) relative to total ERK2 (totERK 42; Mw ≈42 kDa) in bone morphogenetic factor (BMP)4-treated human endothelial kidney (HEK)293 cells transfected with a plasmid encoding human GPC6 (pcGPC6) or vehicle (A-C), n = 3. Glyceraldehyde 3-phosphate dehydrogenase (GAPDH) was used as loading control. Representative immunoblots and quantification of pERK/totERK1 and 2 in BMP4- and non-treated rat cardiac neonatal fibroblasts (NFB), transduced with an adenovirus encoding human GPC6 (AdhGPC6) or empty vector (AdNull; D-F), n = 4. Vinculin was used as loading control. Immunoblots in A and D were run under reducing conditions (+dithiothreitol) revealing the unbound N-terminal domain of GPC6 (GPC6N; Mw ≈35kDa). The full length GPC6 (GPC6FL; Mw ≈62kDa) band represents non-glycanated GPC6 where N- and C-terminal domains are held together by disulfide bonds. Relative mRNA levels of α-smooth muscle actin (ACTA2; G), collagen I (COL1A2; H), collagen III (COL3A1; I), lysyl oxidase (LOX; J) and proliferating cell nuclear antigen (PCNA; K) normalized ribosomal protein L4 (RPL4) in BMP4- and non-treated NFB, transduced with AdhGPC6 or empty vector, n = 6–9 from three separate cell cultures. Migration (%) of BMP4- and non-treated NFB transduced with AdhGPC6 or empty vector, 12–96 h after scratch (L), n = 1–3 images of n = 3–6 wells per condition. Data are presented as mean ± S.E.M. Unpaired Student’s *t-test* (B, C) and one-way ANOVA with Bonferroni post-hoc test (E-L) were used to test for statistical significance. *P<0.05; **P<0.01; group significantly different from vehicle-transfected control, empty vector control or non-stimulated control.

GPC1 and 3 are known to modulate BMP signaling during osteogenesis and renal branching morphogenesis [17, 38] and thus, we investigated whether GPC6 modulated BMP4-mediated signaling pathways in the heart. For overexpression of GPC6, HEK293 cells were transfected with a plasmid encoding full-length human GPC6 (pcGPC6; Fig 5A–5C) and cardiac fibroblasts transduced with an adenovirus encoding full-length human GPC6 (AdhGPC6; Fig 5D–5L). Cells were co-treated with BMP4. Upon BMP4 treatment, increased ERK1 (p44) and 2 (p42) MAPK activation was evidenced by increased phosphorylation in HEK293 cells overexpressing GPC6 compared to vehicle-transfected controls (Fig 5A–5C). Importantly, similar results were found in GPC6 overexpressing NFB, i.e. increased ERK1 and 2 phosphorylation after BMP4 treatment (Fig 5D–5F). BMP4 alone had no effect on ERK1 and 2 activation in NFB. Contrary to in HEK293 cells (S4A–S4C Fig), GPC6 overexpression alone did not affect ERK1/2 phosphorylation in NFB. Thus, in cardiac fibroblasts, GPC6 enhanced BMP4-dependent ERK1/2 activation.

To investigate the modulatory effects of GPC6 on BMP4-dependent ERK1/2 signaling in NFB, we assessed aspects of fibroblast function known to be important in fibrosis. In the AB mouse model, we found positive correlations between GPC6 mRNA levels and mRNA of collagen I (COL1A2; *P*<0.001, R^2^ = 0.44) and collagen III (COL3A1; *P*<0.001, R^2^ = 0.42), supportive of a role for GPC6 in cardiac fibrosis. However, NFB overexpressing GPC6 did not produce higher mRNA levels of the myofibroblast marker gene α-smooth muscle actin (ACTA2; Fig 5G), collagen I (COL1A2; Fig 5H), collagen III (COL3A1; Fig 5I) or the collagen crosslinking enzyme lysyl oxidase (LOX; Fig 5J) with or without BMP4 treatment, compared to controls. Fibroblast proliferation, measured by the expression of proliferating cell nuclear antigen (PCNA; Fig 5K), was also unaffected. Moreover, BMP4 treatment or GPC6 overexpression did not affect the rate of cardiac fibroblast migration (Fig 5L and S5 Fig), as determined from the NFB migration scratch assay.

### Glypican-6 Enhances ERK1/2 Signaling and Hypertrophic Responses in Cultured Cardiomyocytes

**Fig 6:**
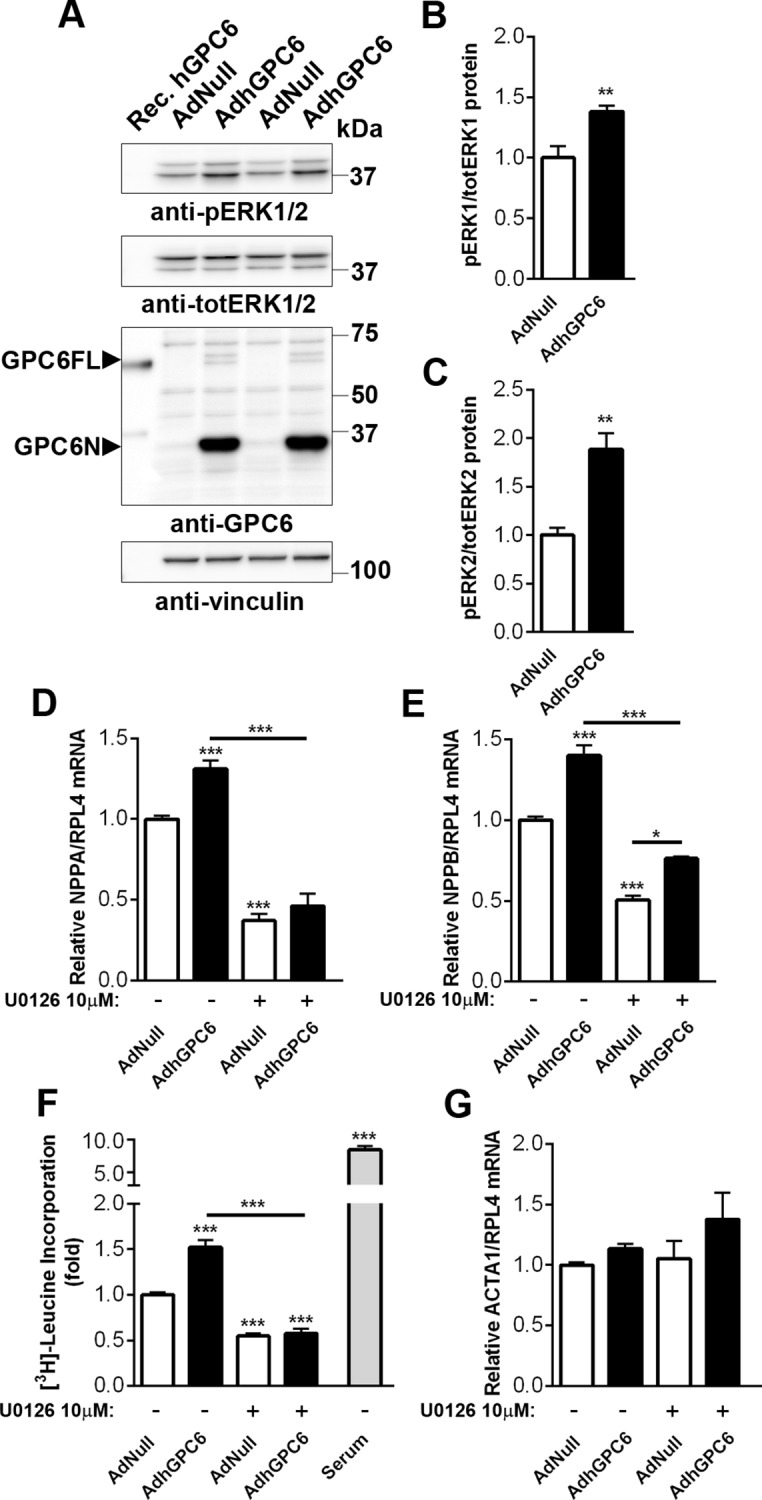
Glypican-6 enhances ERK1/2 signaling and hypertrophic responses in cultured cardiomyocytes. Representative immunoblots and quantification of phospho-extracellular signal-regulated kinase (pERK)1 (pERK 44) relative to total ERK1 (totERK 44; Mw ≈44 kDa) and phospho-ERK2 (pERK 42) relative to total ERK2 (totERK 42; Mw ≈42 kDa) in neonatal rat cardiomyocytes (NCM) transduced with an adenovirus encoding human GPC6 (AdhGPC6) or empty vector (AdNull; A-C), n = 5. Vinculin was used as loading control. Immunoblots in A were run under reducing conditions (+dithiothreitol) revealing the N-terminal domain of GPC6 (GPC6N; ≈35kDa). The full length GPC6 (GPC6FL; Mw ≈62kDa) band represents non-glycanated GPC6 where N- and C-terminal domains are held together by disulfide bonds. Relative mRNA levels of atrial and brain natriuretic peptides (NPPA and NPPB, respectively, D and E) normalized to ribosomal protein L4 (RPL4) in NCM transduced with AdhGPC6 or empty vector, and treated with the dual specificity kinase (MEK1/2) inhibitor U0126 or vehicle control, n = 9–18 from three separate cell cultures. [^3^H] leucine incorporation in NCM transduced with AdhGPC6 or empty vector and treated with U0126 or vehicle control (F), relative to AdNull, non-treated control, n = 6–12. Serum was used as a positive control. Relative mRNA levels of α-skeletal actin (ACTA1, G) normalized to ribosomal protein L4 (RPL4) in NCM transduced with AdhGPC6 or empty vector, and treated with U0126 or vehicle control, n = 8–18 from three separate cell cultures (*t-test* AdNull vs. AdhGPC6, p = 0.0063). Data are presented as mean ± S.E.M. Unpaired Student’s *t-test* (B and C) and one-way ANOVA with Bonferroni post-hoc test (D-G) were used to test for statistical significance. *P<0.05; **P<0.01; ***P<0.001; AdhGPC6-transduced NCM significantly different from empty vector control or U0126-treated groups.

Since glypican-6 was expressed in cardiomyocytes, we transduced NCM with AdhGPC6 and investigated ERK1/2 activation as ERK1/2 are known players in hypertrophic remodeling [39]. Similar to HEK293 cells (see S4A–S4C Fig), cardiomyocytes overexpressing GPC6 displayed increased phosphorylation of ERK1 and 2 (Fig 6A–6C). In contrast to in cardiac fibroblasts (see Fig 5), BMP4 treatment had no additional effect in cardiomyocytes (data not shown). Interestingly, NCM overexpressing GPC6 showed increased mRNA levels of heart failure signature molecules ANP and BNP (Fig 6D and 6E, respectively). To assess whether this response was ERK1/2 dependent, we co-treated cells with the MEK1/2 inhibitor U0126. MEK1/2 is the kinase upstream of the MAPKs ERK1 and 2, phosphorylating and thereby activating them. The ERK1/2 inhibitory effect of U0126 was confirmed in NCM (S6 Fig). The increase in NPPA and NPPB after GPC6 overexpression was attenuated by U0126, suggesting that the GPC6 modulation of NPPA and NPPB expression was ERK1/2-dependent. To assess NCM growth we measured protein synthesis by [^3^H] leucine incorporation. Importantly, cardiomyocytes overexpressing GPC6 exhibited a 1.5-fold higher level of protein synthesis than controls (Fig 6F), and the response was inhibited by co-treatment with U0126. In line with this, NCM overexpressing GPC6 showed a slight increase in mRNA levels of the hypertrophy signature molecule ACTA1 (*t-test* AdNull vs. AdhGPC6, p = 0.0063, Fig 6G). We saw no effect of U0126 on ACTA1 expression. Thus, in cardiomyocytes, increased GPC6 levels induced the expression of heart failure signature molecules and growth through ERK1/2 activation.

## Discussion

This study investigates regulation of the glypican proteoglycan family in experimental and clinical heart failure and demonstrates that GPC6 affects cardiomyocyte signaling and growth. GPC6 showed upregulation during hypertrophic remodeling and cardiac dilatation after pressure overload in mice. Moreover, GPC6 was upregulated in myocardial samples from end-stage heart failure patients. *In vitro* experiments revealed that both cardiac myocytes and fibroblasts produce GPC6. BMP4 stimulated GPC6 expression in cardiac fibroblasts, and GPC6 overexpression potentiated BMP4-induced ERK1/2 activation. In cardiomyocytes, GPC6 overexpression induced hypertrophic responses, including increased protein synthesis and expression of hypertrophic signature genes NPPA, NPPB and ACTA1. ERK1/2 signaling was activated in cardiomyocytes overexpressing GPC6, and by blocking ERK1/2 GPC6-induced hypertrophic responses were attenuated.

GPC1, 3–4 and 6 are known to be expressed in the heart [12, 13, 40, 41], and to our knowledge, this is the first study to explore regulation and roles of glypicans in cardiac remodeling and failure. We found that GPC5 was not expressed in the mouse heart, while GPC1-3 showed minor, and GPC4 no, alterations after pressure overload. GPC6 was up-regulated through initial hypertrophic remodeling and later phases with cardiac dilatation. The isoform-specific regulation of glypicans after pressure overload contrasts alterations reported for a family of transmembrane HS proteoglycans, the syndecans (SDC1-4), where all four isoforms are upregulated in response to pressure overload [8]. Moreover, whereas syndecans are upregulated acutely (e.g. 24 h after AB) and during initial hypertrophic remodeling [8], glypican expression is increased later and persists into dilatation. These findings suggest different roles for glypicans and syndecans HS cell surface proteoglycans during cardiac remodeling and failure.

We focused our studies on GPC6 due to its sustained upregulation after pressure overload. Furthermore, the finding that GPC6 levels in biopsies from patients with end-stage heart failure correlated with reduced EF suggested that GPC6 levels are associated with severity of heart failure. Interestingly, congenital heart defects are reported in patients with the generalized OMOD1 dwarfism syndrome caused by mutations in the GPC6 gene [30], supporting an important role for GPC6 in the heart. OMOD1 is a rare and severe autosomal recessive skeletal dysplasia disorder with dwarfism, facial anomalies and other variable findings [27–29]. Importantly, all affected patients harbor homozygous or compound heterozygous GPC6 mutations predicting absence of a functional protein. This implies that studying GPC6 is highly relevant to understand human disease.

The defects reported in OMOD1 suggest roles for GPC6 associated with cellular growth. GPC6 shares sequence homology with the other glypicans, including conserved cysteine residues and C-terminal serine/glycine GAG chain attachment sites [41]. However, the glypican isoforms do not seem to be redundant since the other glypicans are unable to compensate for the complete loss of GPC6 in OMOD1 [26]. Thus, we hypothesized that the increased GPC6 expression observed in the LV after pressure overload regulates hypertrophic remodeling in the heart. Indeed, our results from cultured cardiomyocytes overexpressing GPC6 suggested that GPC6 induces hypertrophic growth, shown by protein synthesis assays and expression of signature genes.

The hypertrophic response induced in cultured cardiomyocytes upon GPC6 overexpression was accompanied by activation of ERK1/2 signaling. ERK1/2 are known to be central regulators of pathological hypertrophy, and act as downstream mediators of extracellular stimuli [42]. Inhibition of ERK1/2 abrogates neurohumoral-induced hypertrophy in cardiomyocytes [43, 44], and we observed suppression of GPC6-induced hypertrophic response after pharmacological inhibition of ERK1/2. Glypicans typically function as cell surface regulators of ligand-receptor interactions [14], and whether GPC6 is a direct or indirect activator of the MAPK cascade leading to hypertrophic growth remains uncertain. Nevertheless, our results clearly show that enhanced GPC6 expression in cardiomyocytes induces hypertrophic responses in an ERK1/2-dependent manner. This mechanism is likely also to occur during *in vivo* cardiac hypertrophy. During cardiac remodeling and failure in response to pathological stimuli, the fetal gene program is activated [45]. GPC6 plays a pivotal role in embryonic development [41], and the observed upregulation after pressure overload therefore could be part of the transcriptional program triggered whereby genes important for fetal heart development are re-expressed.

We show that BMP4, a member of the TGF superfamily known to influence embryonic cardiac structure [46], is a regulator of GPC6 expression in cardiac fibroblasts. To our knowledge, our study is the first to investigate the relationship between GPC6 and BMPs in the heart. BMP4 was recently found to regulate the degree of hypertrophy and fibrosis after pressure overload and ANGII infusion in rodents [47]. We observed enhanced ERK1/2 activation after BMP4 treatment in HEK293 cells and cardiac fibroblasts overexpressing GPC6. This points to a GPC6-BMP4 interaction, resulting in augmented BMP receptor activation, and possibly a positive feedback loop as BMP4 also enhanced GPC6 expression. However, we did not observe alterations in any markers of fibroblast activation, despite enhanced downstream signaling after BMP4 stimulation. This does not exclude the possibility that GPC6 affects cellular processes in fibroblasts or fibrosis, and the consequences of the observed ERK1/2 activation at this point remain unknown.

Interestingly, when overexpressing GPC6 in primary cardiac cell cultures, we found a difference in GPC6 HS glycanation in fibroblasts and cardiomyocytes, evident by the different size of the proteoglycan being expressed from the same vector. Heterogeneity is a hallmark of HS GAG chains, and regulation of HS GAG chain length and composition adds another level of complexity to understanding proteoglycan biology [48]. Glycomic profiling of HS GAG chains in rodent tissues revealed unique HS GAG composition in different organs [49, 50], and even cells of the same origin can express distinct HS GAG chains in different disease states, as seen in cancerous versus non-cancerous hepatocytes and lethal versus non-lethal breast cancer cells [51, 52]. The functional consequences of different HS GAG composition are altered proteoglycan interactome, as ligands like FGF and the FGF receptor show differential affinity for HS that display specific sulfatation patterns [53, 54]. In addition, the difference in GPC6 HS GAG chain size likely reflects distinct functional attributes in cardiomyocytes and fibroblasts, and we speculate that this could underlie the differences in MAPK signaling response after BMP4 treatment.

In summary, we show that glypican-6 is upregulated in experimental and clinical heart failure. Both cardiac fibroblasts and myocytes produce GPC6 in the heart. Adenoviral overexpression of GPC6 in cardiomyocytes induced hypertrophic growth responses through activation of ERK1/2 MAPK signaling. Collectively, our data suggest that GPC6 enhances hypertrophic signaling during pressure overload-induced cardiac remodeling.

## Supporting Information

S1 Fig Methanol precipitation and heparan sulfate digestion was used to quantify glypican-6 protein in mouse left ventricular tissue. Proteoglycans in left ventricular (LV) tissue protein lysates from mice were methanol (MetOH) precipitated prior to enzymatic heparan sulfate (HS) digestion [8, 36]. HS digestion results in a HS-neo epitope on proteoglycans recognized by the Δ3G10-antibody. Immunoblotting analyzed under reducing conditions (+dithiothreitol) using the Δ3G10 antibody revealed successful enzymatic digestion of left ventricular proteoglycans from mice subjected to aortic banding (AB) or sham-operation for one week. The ≈62kDa protein band likely corresponds to full-length GPC6 without glycanation (GPC6FL) [33], seen in Fig 1H.
(TIF)

S2 Fig Glypican-6 protein chemistry showing the expected immunoblotting migration pattern in cardiac cell samples treated under reducing and non-reducing conditions. Protein chemistry to confirm reported GPC6 immunoblot protein bands [33, 37] in rat cardiac fibroblasts (NFB; A) and myocytes (NCM; B) transduced with an adenovirus serotype 5 encoding human GPC6 (AdhGPC6) or empty vector (AdNull), using a polyclonal antibody with epitopes in the N-terminal domain of GPC6 [33]. NFB and NCM protein lysates run under non-reducing conditions (-dithiothreitol (DTT)) revealed the full-length glycanated protein (GPC6glyc, Mw >150 kDa) carrying four heparan sulfate (HS) glycosaminoglycan (GAG) chains and the N-terminal GPC6 domain (GPC6N, Mw ≈35kDa). The same lysates run under reducing condition (+ DTT) showed loss of GPC6glyc bands and an enhanced GPC6N signal due to reduction of the disulfide bonds connecting the N- and C-terminal domains. The full length GPC6 (GPC6FL; Mw ≈62kDa) band present in A and B represented non-glycanated GPC6 where C- and N-terminal domains are held together by the disulfide bonds. Non-glycanated recombinant human GPC6 (Rec.hGPC6) was used as a positive control for the antibody and vinculin was used for loading control. Immunoblot of GPC6 in methanol-precipitated and heparitinase-digested [8, 36] protein lysates from NFB transduced with AdhGPC6 or AdNull run under non-reducing (-DTT) and reducing (+ DTT) conditions (C). Non-digested samples under reducing and non-reducing conditions were included as controls for successful HS digestion. By digesting the HS GAG chains before separation under non-reducing conditions, the GPC6glyc bands is no longer present and the deglycanated GPC6FL appears at ≈62kDa. The same samples run under reducing conditions completely lack the GPC6glyc and GPC6FL bands, and show enhanced GPC6N signal. Thus, preparation of samples under reducing conditions alters the migration pattern of GPC6 by reducing the disulfide bridges connecting the N- and C-terminal domains together. Conclusively, these results show that GPC6N abundance represents full-length GPC6 levels when samples are prepared under reducing conditions, and we therefore used GPC6N levels as a read-out of total GPC6 protein levels in cells throughout our study. D, schematic illustration of GPC6glyc, GPC6FL and GPC6N from left to right (modified from [15]).
(TIF)

S3 Fig Regulation of glypican-6 expression in neonatal rat cardiac cells. GPC6 mRNA in neonatal rat cardiac fibroblasts (NFB) after 24 h treatment with angiotensin (ANG)II, norepinephrine (NE), transforming growth factor (TGF)β1, basic fibroblast growth factor (FGF2), platelet-derived growth factor (PDGF)-BB, tumor necrosis factor (TNF)α, interleukin (IL)-1β or IL-18 (A), n = 3–12. GPC6 mRNA in neonatal rat cardiomyocytes (NCM) after 24 h treatment with ANGII, bone morphogenetic protein (BMP)4, NE, TGFβ1, TNFα, IL-1β or IL-18 (B), n = 3–12. Data are presented as mean ± S.E.M. Unpaired Student’s *t-test* were used to test for statistical significance. *P<0.05; **P<0.01; ***P<0.001; treated group different from control.
(TIF)

S4 Fig Glypican-6 enhances ERK1/2 signaling in cultured HEK293 cells. Representative immunoblots and quantification of phospho-extracellular signal-regulated kinase (pERK)1 (pERK 44) and total ERK1 (totERK 44; ≈44 kDa), and phospho-ERK2 (pERK 42) and ERK2 (totERK 42; ≈42 kDa) in human endothelial kidney (HEK)293 cells transfected with a plasmid encoding human GPC6 (pcGPC6) or vehicle (A-C), analyzed under reducing conditions (+dithiothreitol), n = 9–10. Glyceraldehyde 3-phosphate dehydrogenase (GAPDH) was used as loading control. Data are presented as mean ± S.E.M. Unpaired Student’s *t-test* was used to test for statistical significance. *P<0.05; group significantly different from vehicle-transfected control.
(TIF)

S5 Fig Migration analysis of cultured neonatal rat cardiac fibroblasts. Representative images of neonatal rat fibroblasts (NFB) transduced with an adenovirus encoding human GPC6 (AdhGPC6) or empty vector (AdNull), with or without co-treatment with bone morphogenetic protein (BMP)4. Images show cells at time 0 after a vertical scratch has been made and the same area after 96 h of cell migration. N = 3–6 wells per experimental treatment with n = 1–3 areas per well. Average data are shown in Fig 5L.
(TIF)

S6 Fig Inhibition of ERK1/2 signaling in cultured cardiomyocytes. Representative immunoblots of phospho-extracellular signal-regulated kinase (pERK)1 (pERK 44) and total ERK1 (totERK 44; ≈44 kDa), and phospho-ERK2 (pERK 42) and ERK2 (totERK 42; ≈42 kDa) in rat cardiac neonatal cardiomyocytes (NCM) transduced with an adenovirus encoding human GPC6 (AdhGPC6) and in non-transduced controls, analyzed under reducing conditions (+dithiothreitol), n = 3. The dual specificity kinase (MEK1/2) inhibitor U0126 was used to inhibit ERK1/2 activation by phosphorylation. Vinculin was used as loading control.
(TIF)

## References

[1] Bui AL, Horwich TB, Fonarow GC. Epidemiology and risk profile of heart failure. Nat Rev Cardiol. 2011;8(1):30–41. 10.1038/nrcardio.2010.165 21060326 PMC3033496

[2] Dunlay SM, Weston SA, Jacobsen SJ, Roger VL. Risk factors for heart failure: a population-based case-control study. Am J Med. 2009;122(11):1023–8. 10.1016/j.amjmed.2009.04.022 19854330 PMC2789475

[3] Zile MR, Brutsaert DL. New concepts in diastolic dysfunction and diastolic heart failure: part II: causal mechanisms and treatment. Circulation. 2002;105(12):1503–8. 11914262 10.1161/hc1202.105290

[4] Kaye DM, Krum H. Drug discovery for heart failure: a new era or the end of the pipeline? Nat Rev Drug Discov. 2007;6(2):127–39. 10.1038/nrd2219 17268484

[5] Finsen AV, Lunde IG, Sjaastad I, Østli EK, Lyngra M, Jarstadmarken HO, et al Syndecan-4 is essential for development of concentric myocardial hypertrophy via stretch-induced activation of the calcineurin-NFAT pathway. PLoS ONE. 2011;6(12):e28302 10.1371/journal.pone.0028302 22164265 PMC3229559

[6] Herum KM, Lunde IG, Skrbic B, Florholmen G, Behmen D, Sjaastad I, et al Syndecan-4 signaling via NFAT regulates extracellular matrix production and cardiac myofibroblast differentiation in response to mechanical stress. J Mol Cell Cardiol. 2013;54:73–81. 10.1016/j.yjmcc.2012.11.006 23178899

[7] Herum KM, Lunde IG, Skrbic B, Louch WE, Hasic A, Boye S, et al Syndecan-4 is a key determinant of collagen cross-linking and passive myocardial stiffness in the pressure-overloaded heart. Cardiovasc Res. 2015;106(2):217–26. 10.1093/cvr/cvv002 25587045

[8] Strand ME, Herum KM, Rana ZA, Skrbic B, Askevold ET, Dahl CP, et al Innate immune signaling induces expression and shedding of the heparan sulfate proteoglycan syndecan-4 in cardiac fibroblasts and myocytes, affecting inflammation in the pressure-overloaded heart. FEBS J. 2013;280(10):2228–47. 10.1111/febs.12161 23374111

[9] Finsen AV, Woldbaek PR, Li J, Wu J, Lyberg T, Tonnessen T, et al Increased syndecan expression following myocardial infarction indicates a role in cardiac remodeling. Physiol Genomics. 2004;16(3):301–8. 10.1152/physiolgenomics.00144.2002 14625378

[10] Bernfield M, Kokenyesi R, Kato M, Hinkes MT, Spring J, Gallo RL, et al Biology of the syndecans: a family of transmembrane heparan sulfate proteoglycans. Annu Rev Cell Biol. 1992;8(1):365–93. 1335744 10.1146/annurev.cb.08.110192.002053

[11] Filmus J, Capurro M, Rast J. Glypicans. Genome Biol. 2008;9(5):224 10.1186/gb-2008-9-5-224 18505598 PMC2441458

[12] Traister A, Shi W, Filmus J. Mammalian Notum induces the release of glypicans and other GPI-anchored proteins from the cell surface. Biochem J. 2008;410(3):503–11. 10.1042/BJ20070511 17967162

[13] Paine-Saunders S, Viviano BL, Saunders S. GPC6, a novel member of the glypican gene family, encodes a product structurally related to GPC4 and is colocalized with GPC5 on human chromosome 13. Genomics. 1999;57(3):455–8. 10.1006/geno.1999.5793 10329016

[14] Fico A, Maina F, Dono R. Fine-tuning of cell signaling by glypicans. Cell Mol Life Sci. 2011;68(6):923–9. 10.1007/s00018-007-7471-6 18087675 PMC11114805

[15] Filmus J, Selleck SB. Glypicans: proteoglycans with a surprise. J Clinl Invest. 2001;108(4):497–501. 10.1172/JCI13712 PMC209407 11518720

[16] Paine-Saunders S, Viviano BL, Zupicich J, Skarnes WC, Saunders S. Glypican-3 controls cellular responses to Bmp4 in limb patterning and skeletal development. Dev Biol. 2000;225(1):179–87 10.1006/dbio.2000.9831 10964473

[17] Dwivedi PP, Grose RH, Filmus J, Hii CS, Xian CJ, Anderson PJ, et al Regulation of bone morphogenetic protein signalling and cranial osteogenesis by Gpc1 and Gpc3. Bone. 2013;55(2):367–76. 10.1016/j.bone.2013.04.013 23624389

[18] Midorikawa Y, Ishikawa S, Iwanari H, Imamura T, Sakamoto H, Miyazono K, et al Glypican-3, overexpressed in hepatocellular carcinoma, modulates FGF2 and BMP-7 signaling. Int J Cancer. 2003;103(4):455–65. 10.1002/ijc.10856 12478660

[19] Hartwig S, Hu M-C, Cella C, Piscione T, Filmus J, Rosenblum ND. Glypican-3 modulates inhibitory Bmp2-Smad signaling to control renal development in vivo. Mech Dev. 2005;122(7–8):928–38. 10.1016/j.mod.2005.03.007 15925496

[20] Hoffmann A, Gross G. BMP signaling pathways in cartilage and bone formation. Crit Rev Eukaryot Gene Expr. 2001;11(1–3):23–45. 11693963

[21] Bleuming SA, He XC, Kodach LL, Hardwick JC, Koopman FA, ten Kate FJ, et al Bone morphogenetic protein signaling suppresses tumorigenesis at gastric epithelial transition zones in mice. Cancer Res. 2007;67(17):8149–55. 10.1158/0008-5472.CAN-06-4659 17804727

[22] Langenfeld EM, Bojnowski J, Perone J, Langenfeld J. Expression of bone morphogenetic proteins in human lung carcinomas. Ann Thorac Surg. 2005;80(3):1028–32. 10.1016/j.athoracsur.2005.03.094 16122479

[23] Miyazono K, Kamiya Y, Morikawa M. Bone morphogenetic protein receptors and signal transduction. J Biochem. 2010;147(1):35–51. 10.1093/jb/mvp148 19762341

[24] Wang RN, Green J, Wang Z, Deng Y, Qiao M, Peabody M, et al Bone Morphogenetic Protein (BMP) signaling in development and human diseases. Genes Dis. 2014;1(1):87–105. 10.1016/j.gendis.2014.07.005 25401122 PMC4232216

[25] Pilia G, Hughes-Benzie RM, MacKenzie A, Baybayan P, Chen EY, Huber R, et al Mutations in GPC3, a glypican gene, cause the Simpson-Golabi-Behmel overgrowth syndrome. Nat Genet. 1996;12(3):241–7. 10.1038/ng0396-241 8589713

[26] Campos-Xavier AB, Martinet D, Bateman J, Belluoccio D, Rowley L, Tan TY, et al Mutations in the heparan-sulfate proteoglycan glypican 6 (GPC6) impair endochondral ossification and cause recessive omodysplasia. Am J Hum Genet. 2009;84(6):760–70. 10.1016/j.ajhg.2009.05.002 19481194 PMC2694977

[27] Albano LM, Oliveira LAN, Bertola DR, Mazzu JF, Kim CA. Omodysplasia: the first reported Brazilian case. Clinics. 2007;62:531–4. 17823719 10.1590/s1807-59322007000400023

[28] Borochowitz Z, Barak M, Hershkowitz S. Familial congenital micromelic dysplasia with dislocation of radius and distinct face: a new skeletal dysplasia syndrome. Am J Med Genet. 1991;39(1):91–6. 10.1002/ajmg.1320390120 1867270

[29] Elcioglu NH, Gustavson KH, Wilkie AO, Yuksel-Apak M, Spranger JW. Recessive omodysplasia: five new cases and review of the literature. Pediatr Radiol. 2004;34(1):75–82. 10.1007/s00247-003-1064-9 14566439

[30] Baxová A, Maroteaux P, Barošová J, Netriová I. Parental consanguinity in two sibs with omodysplasia. Am J Med Genet. 1994;49(3):263–5. 10.1002/ajmg.1320490303 8209882

[31] Lin AE, Neri G, Hughes-Benzie R, Weksberg R. Cardiac anomalies in the Simpson-Golabi-Behmel syndrome. Am J Med Genet. 1999;83(5):378–81. 10232747

[32] Skrbic B, Engebretsen KV, Strand ME, Lunde IG, Herum KM, Marstein HS, et al Lack of collagen VIII reduces fibrosis and promotes early mortality and cardiac dilatation in pressure overload in mice. Cardiovasc Res. 2015;106(1):32–42. 10.1093/cvr/cvv041 25694587

[33] Capurro M, Martin T, Shi W, Filmus J. Glypican-3 binds to Frizzled and plays a direct role in the stimulation of canonical Wnt signaling. J Cell Sci. 2014;127(7):1565–75. 24496449 10.1242/jcs.140871

[34] Lunde IG, Kvaløy H, Austbø B, Christensen G, Carlson CR. Angiotensin II and norepinephrine activate specific calcineurin-dependent NFAT transcription factor isoforms in cardiomyocytes. J Appl Physiol. 2011;111(5):1278–89. 10.1152/japplphysiol.01383.2010 21474694

[35] Strand ME, Aronsen JM, Braathen B, Sjaastad I, Kvaløy H, Tønnessen T, et al Shedding of syndecan-4 promotes immune cell recruitment and mitigates cardiac dysfunction after lipopolysaccharide challenge in mice. J Mol Cell Cardiol. 2015;88:133–44. 10.1016/j.yjmcc.2015.10.003 26449522

[36] Burbach BJ, Friedl A, Mundhenke C, Rapraeger AC. Syndecan-1 accumulates in lysosomes of poorly differentiated breast carcinoma cells. Matrix Biol. 2003;22(2):163–77. 12782143 10.1016/s0945-053x(03)00009-x

[37] Capurro MI, Shi W, Sandal S, Filmus J. Processing by convertases is not required for glypican-3-induced stimulation of hepatocellular carcinoma growth. J Biol Chem. 2005;280(50):41201–6. 10.1074/jbc.M507004200 16227623

[38] Grisaru S, Cano-Gauci D, Tee J, Filmus J, Rosenblum ND. Glypican-3 modulates BMP- and FGF-mediated effects during renal branching morphogenesis. Dev Biol. 2001;231(1):31–46. 10.1006/dbio.2000.0127 11180950

[39] Ruppert C, Deiss K, Herrmann S, Vidal M, Oezkur M, Gorski A, et al Interference with ERK(Thr188) phosphorylation impairs pathological but not physiological cardiac hypertrophy. Proc Natl Acad Sci U S A. 2013;110(18):7440–5. 10.1073/pnas.1221999110 23589880 PMC3645583

[40] Strunz CMC, Matsuda M, Salemi VMC, Nogueira A, Mansur AP, Cestari IN, et al Changes in cardiac heparan sulfate proteoglycan expression and streptozotocin-induced diastolic dysfunction in rats. Cardiovasc Diabetol. 2011;10:35 10.1186/1475-2840-10-35 21518435 PMC3100243

[41] Veugelers M, De Cat B, Ceulemans H, Bruystens A-M, Coomans C, Dürr J, et al Glypican-6, a new member of the glypican family of cell surface heparan sulfate proteoglycans. J Biol Chem. 1999;274(38):26968–77. 10480909 10.1074/jbc.274.38.26968

[42] Zhang W, Elimban V, Nijjar MS, Gupta SK, Dhalla NS. Role of mitogen-activated protein kinase in cardiac hypertrophy and heart failure. Exp Clin Cardiol. 2003;8(4):173–83. 19649217 PMC2719157

[43] Aoki H, Richmond M, Izumo S, Sadoshima J. Specific role of the extracellular signal-regulated kinase pathway in angiotensin II-induced cardiac hypertrophy in vitro. Biochem J. 2000;347 Pt 1:275–84. 10727428 PMC1220957

[44] Yue T-L, Gu J-L, Wang C, Reith AD, Lee JC, Mirabile RC, et al Extracellular Signal-regulated Kinase plays an essential role in hypertrophic agonists, Endothelin-1 and Phenylephrine-induced cardiomyocyte hypertrophy. J Biol Chem. 2000;275(48):37895–901. 10.1074/jbc.M007037200 10984495

[45] Dirkx E, da Costa Martins PA, De Windt LJ. Regulation of fetal gene expression in heart failure. Biochim Biophys Acta Mol Basis Dis. 2013;1832(12):2414–24. 10.1016/j.bbadis.2013.07.023 24036209

[46] McCulley DJ, Kang J-O, Martin JF, Black BL. BMP4 is required in the anterior heart field and its derivatives for endocardial cushion remodeling, outflow tract septation, and semilunar valve development. Dev Dyn. 2008;237(11):3200–9. 10.1002/dvdy.21743 18924235 PMC2728547

[47] Sun B, Huo R, Sheng Y, Li Y, Xie X, Chen C, et al Bone morphogenetic protein-4 mediates cardiac hypertrophy, apoptosis, and fibrosis in experimentally pathological cardiac hypertrophy. Hypertension. 2013;61(2):352–60. 10.1161/HYPERTENSIONAHA.111.00562 23248151

[48] Sarrazin S, Lamanna WC, Esko JD. Heparan sulfate proteoglycans. CSH Perspect Biol. 2011;3(7). 10.1101/cshperspect.a004952 PMC3119907 21690215

[49] Shi X, Zaia J. Organ-specific heparan sulfate structural phenotypes. J Biol Chem. 2009;284(18):11806–14. 10.1074/jbc.M809637200 19244235 PMC2673249

[50] Warda M, Toida T, Zhang F, Sun P, Munoz E, Xie J, et al Isolation and characterization of heparan sulfate from various murine tissues. Glycoconj J. 2006;23(0):555–63. 17006646 10.1007/s10719-006-7668-1 PMC4140570

[51] Weyers A, Yang B, Yoon DS, Park J-H, Zhang F, Lee KB, et al A structural analysis of glycosaminoglycans from lethal and nonlethal breast cancer tissues: toward a novel class of theragnostics for personalized medicine in oncology? OMICS. 2012;16(3):79–89. 10.1089/omi.2011.0102 22401653 PMC3300064

[52] Tátrai P, Egedi K, Somorácz Á, van Kuppevelt TH, ten Dam G, Lyon M, et al Quantitative and qualitative alterations of heparan sulfate in fibrogenic liver diseases and hepatocellular cancer. J Histochem Cytochem. 2010;58(5):429–41. 10.1369/jhc.2010.955161 20124094 PMC2857815

[53] Jastrebova N, Vanwildemeersch M, Rapraeger AC, Giménez-Gallego G, Lindahl U, Spillmann D. Heparan sulfate-related oligosaccharides in ternary complex formation with fibroblast growth factors 1 and 2 and their receptors. J Biol Chem. 2006;281(37):26884–92. 10.1074/jbc.M600806200 16807244

[54] Pye DA, Vives RR, Turnbull JE, Hyde P, Gallagher JT. Heparan sulfate oligosaccharides require 6-O-sulfation for promotion of basic fibroblast growth factor mitogenic activity. J Biol Chem. 1998;273(36):22936–42. 9722514 10.1074/jbc.273.36.22936

