# Supplementary figures and images for: The Heparan Sulfate Proteoglycan Glypican-6 Is Upregulated in the Failing Heart, and Regulates Cardiomyocyte Growth through ERK1/2 Signaling
Source: PLoS One. 2016 Oct 21;11(10):e0165079. doi: 10.1371/journal.pone.0165079 (PMC5074531; doi:10.1371/journal.pone.0165079)

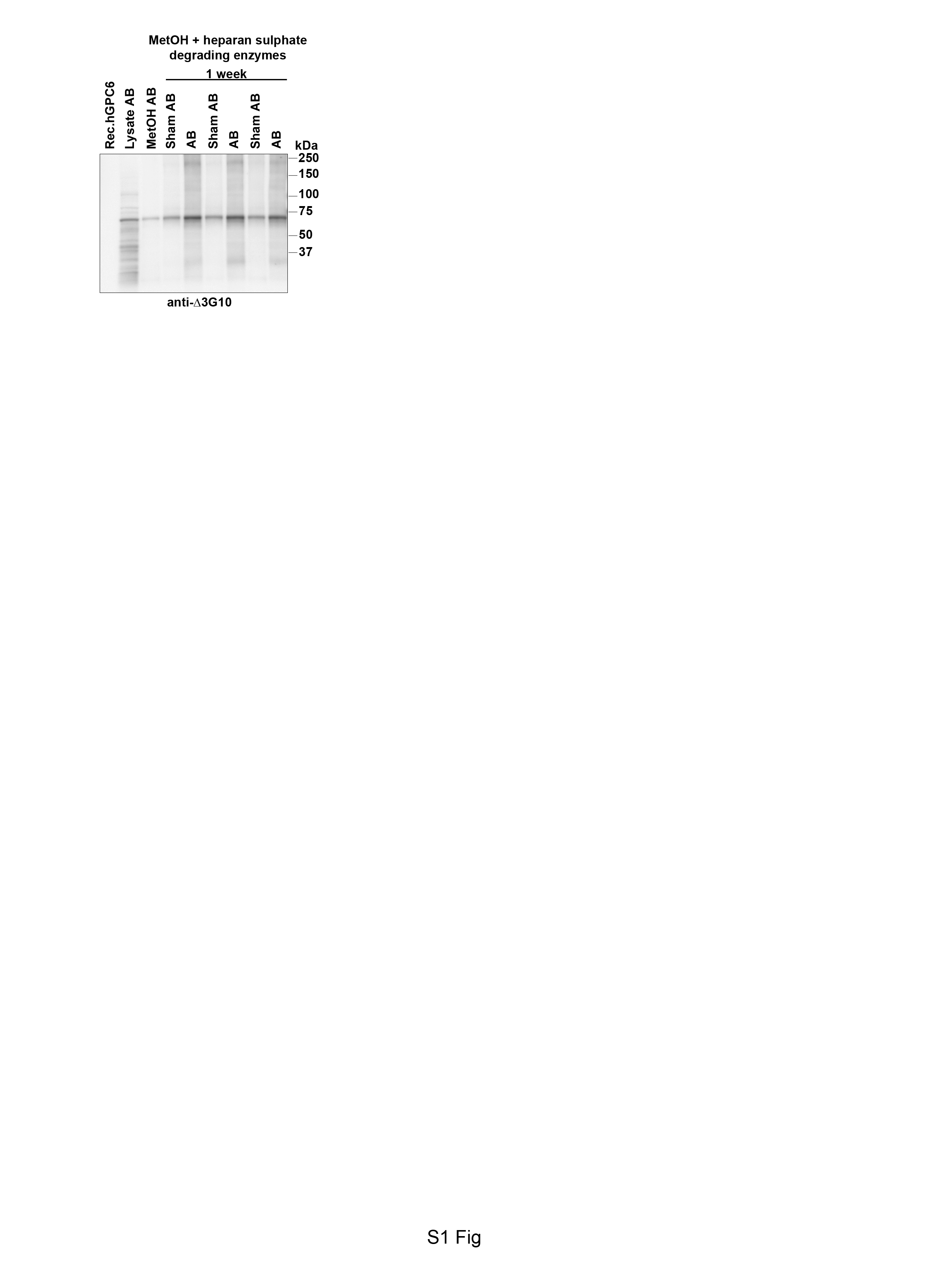

Supplement: S1 Fig — Proteoglycans in left ventricular (LV) tissue protein lysates from mice were methanol (MetOH) precipitated prior to enzymatic heparan sulfate (HS) digestion [8, 36]. HS digestion results in a HS-neo epitope on proteoglycans recognized by the Δ3G10-antibody. Immunoblotting analyzed under reducing conditions (+dithiothreitol) using the Δ3G10 antibody revealed successful enzymatic digestion of left ventricular proteoglycans from mice subjected to aortic banding (AB) or sham-operation for one week. The ≈62kDa protein band likely corresponds to full-length GPC6 without glycanation (GPC6FL) [33], seen in Fig 1H. (TIF) [file pone.0165079.s001.tif]

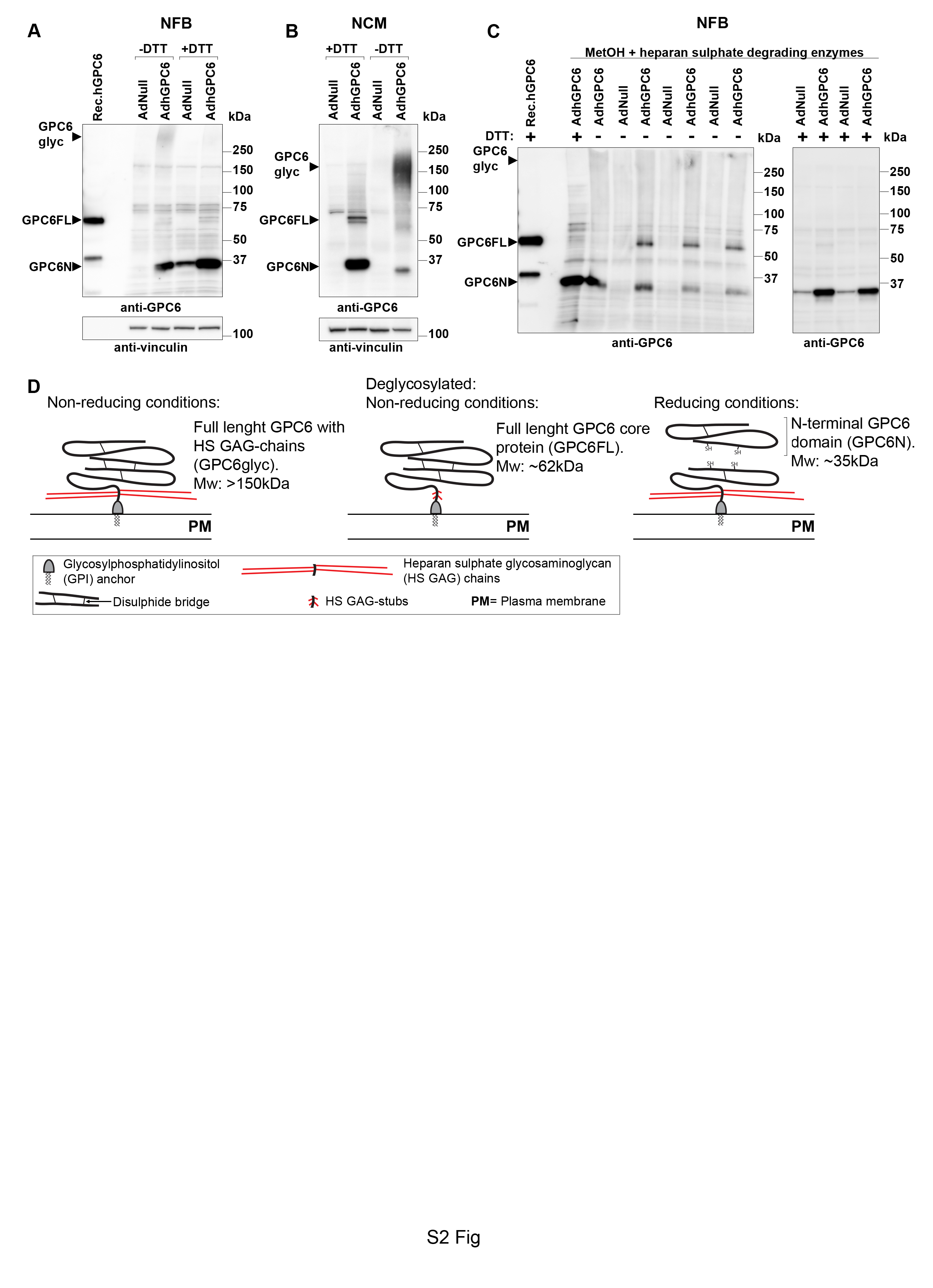

Supplement: S2 Fig — Protein chemistry to confirm reported GPC6 immunoblot protein bands [33, 37] in rat cardiac fibroblasts (NFB; A) and myocytes (NCM; B) transduced with an adenovirus serotype 5 encoding human GPC6 (AdhGPC6) or empty vector (AdNull), using a polyclonal antibody with epitopes in the N-terminal domain of GPC6 [33]. NFB and NCM protein lysates run under non-reducing conditions (-dithiothreitol (DTT)) revealed the full-length glycanated protein (GPC6glyc, Mw >150 kDa) carrying four heparan sulfate (HS) glycosaminoglycan (GAG) chains and the N-terminal GPC6 domain (GPC6N, Mw ≈35kDa). The same lysates run under reducing condition (+ DTT) showed loss of GPC6glyc bands and an enhanced GPC6N signal due to reduction of the disulfide bonds connecting the N- and C-terminal domains. The full length GPC6 (GPC6FL; Mw ≈62kDa) band present in A and B represented non-glycanated GPC6 where C- and N-terminal domains are held together by the disulfide bonds. Non-glycanated recombinant human GPC6 (Rec.hGPC6) was used as a positive control for the antibody and vinculin was used for loading control. Immunoblot of GPC6 in methanol-precipitated and heparitinase-digested [8, 36] protein lysates from NFB transduced with AdhGPC6 or AdNull run under non-reducing (-DTT) and reducing (+ DTT) conditions (C). Non-digested samples under reducing and non-reducing conditions were included as controls for successful HS digestion. By digesting the HS GAG chains before separation under non-reducing conditions, the GPC6glyc bands is no longer present and the deglycanated GPC6FL appears at ≈62kDa. The same samples run under reducing conditions completely lack the GPC6glyc and GPC6FL bands, and show enhanced GPC6N signal. Thus, preparation of samples under reducing conditions alters the migration pattern of GPC6 by reducing the disulfide bridges connecting the N- and C-terminal domains together. Conclusively, these results show that GPC6N abundance represents full-length GPC6 levels when samples are prepared u [file pone.0165079.s002.tif]

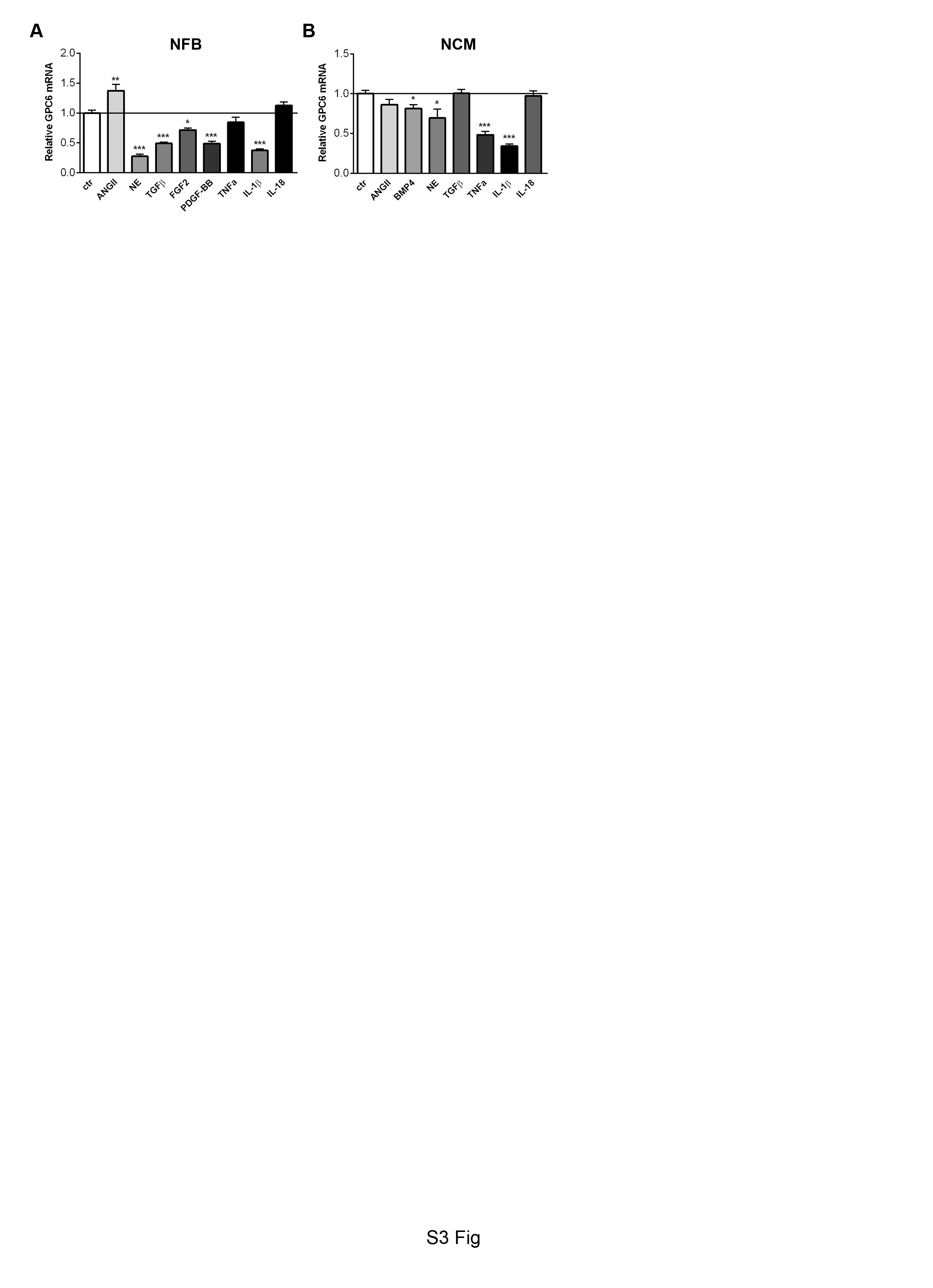

Supplement: S3 Fig — GPC6 mRNA in neonatal rat cardiac fibroblasts (NFB) after 24 h treatment with angiotensin (ANG)II, norepinephrine (NE), transforming growth factor (TGF)β1, basic fibroblast growth factor (FGF2), platelet-derived growth factor (PDGF)-BB, tumor necrosis factor (TNF)α, interleukin (IL)-1β or IL-18 (A), n = 3–12. GPC6 mRNA in neonatal rat cardiomyocytes (NCM) after 24 h treatment with ANGII, bone morphogenetic protein (BMP)4, NE, TGFβ1, TNFα, IL-1β or IL-18 (B), n = 3–12. Data are presented as mean ± S.E.M. Unpaired Student’s t-test were used to test for statistical significance. *P<0.05; **P<0.01; ***P<0.001; treated group different from control. (TIF) [file pone.0165079.s003.tif]

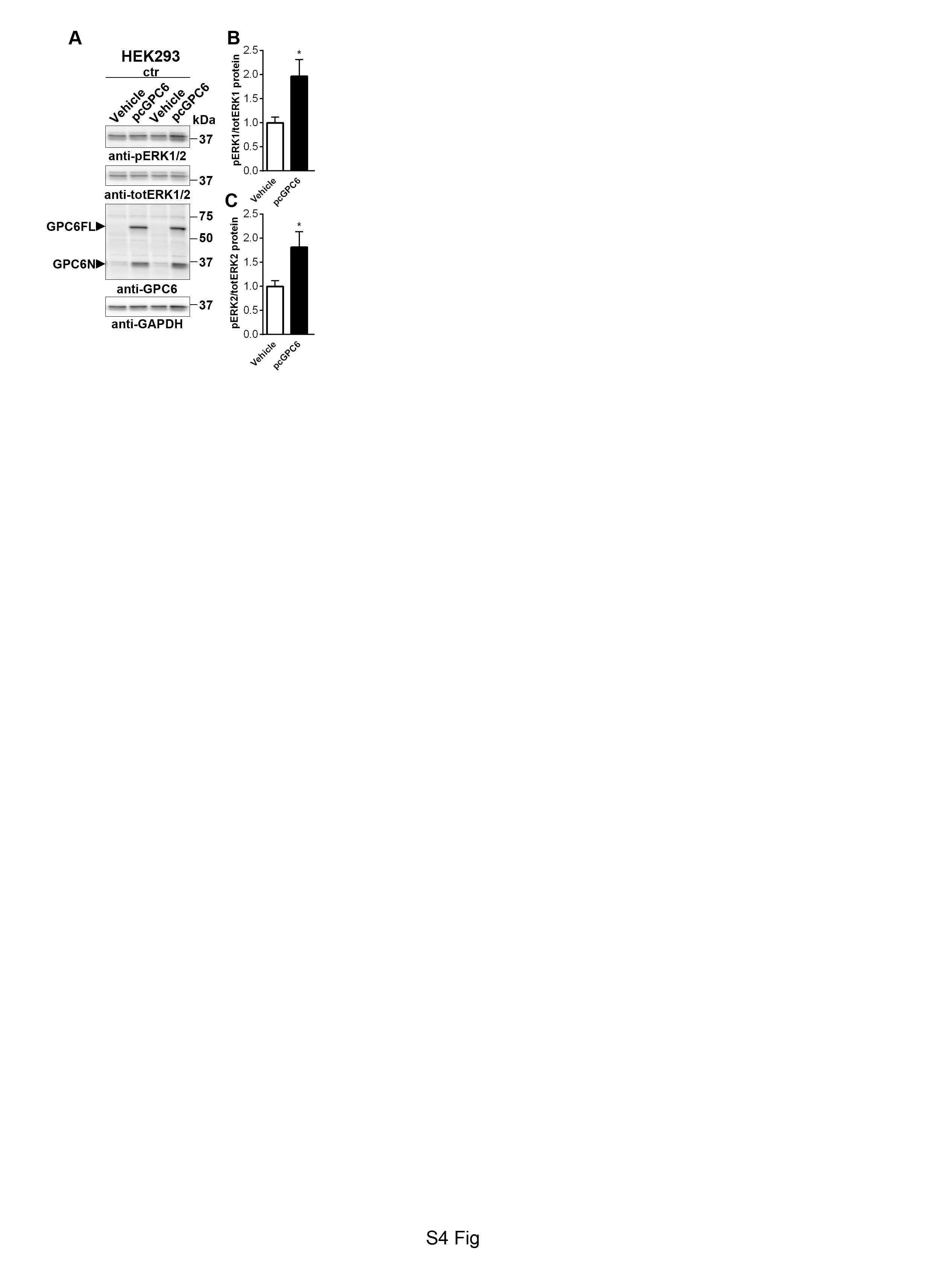

Supplement: S4 Fig — Representative immunoblots and quantification of phospho-extracellular signal-regulated kinase (pERK)1 (pERK 44) and total ERK1 (totERK 44; ≈44 kDa), and phospho-ERK2 (pERK 42) and ERK2 (totERK 42; ≈42 kDa) in human endothelial kidney (HEK)293 cells transfected with a plasmid encoding human GPC6 (pcGPC6) or vehicle (A-C), analyzed under reducing conditions (+dithiothreitol), n = 9–10. Glyceraldehyde 3-phosphate dehydrogenase (GAPDH) was used as loading control. Data are presented as mean ± S.E.M. Unpaired Student’s t-test was used to test for statistical significance. *P<0.05; group significantly different from vehicle-transfected control. (TIF) [file pone.0165079.s004.tif]

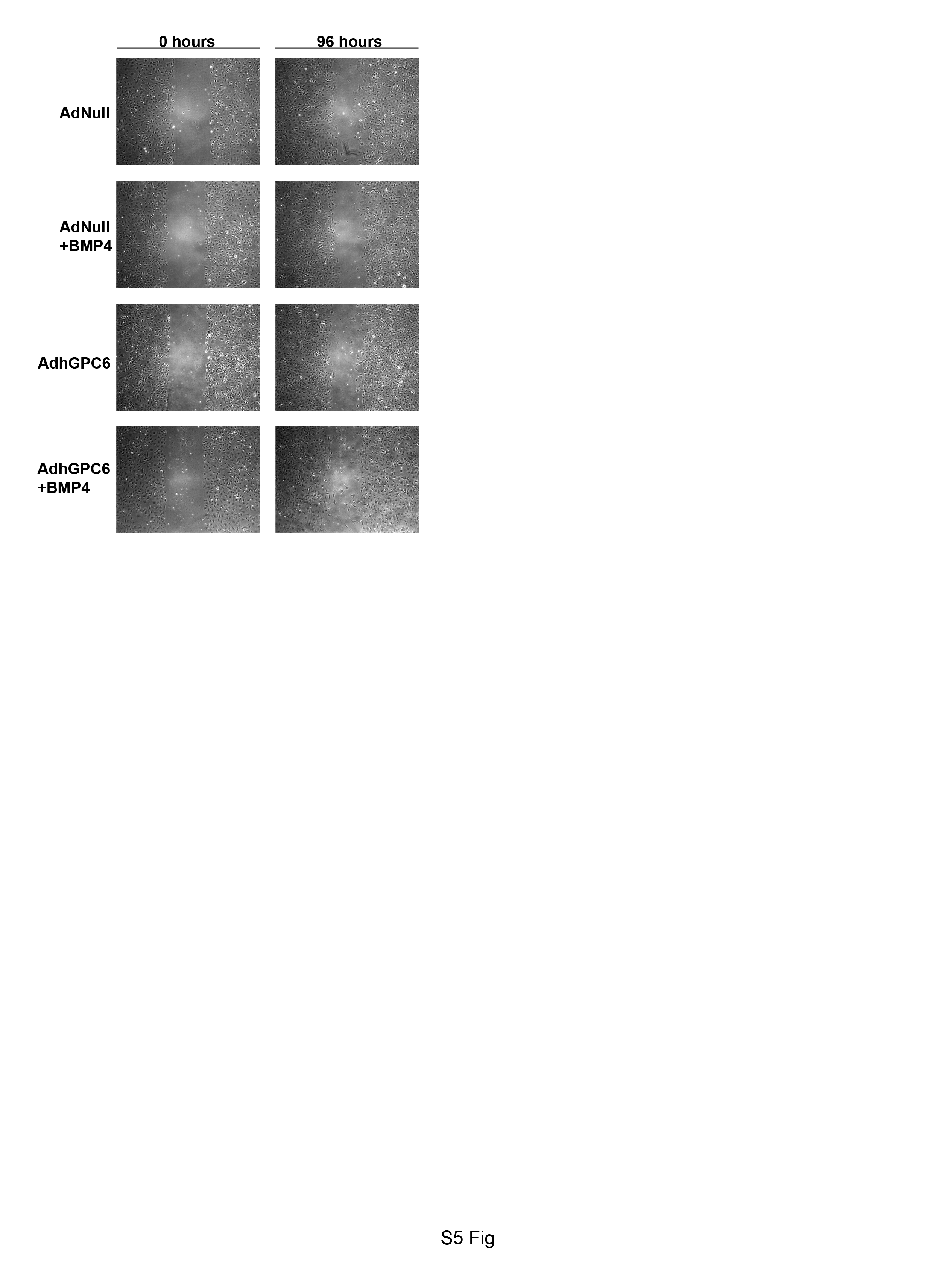

Supplement: S5 Fig — Representative images of neonatal rat fibroblasts (NFB) transduced with an adenovirus encoding human GPC6 (AdhGPC6) or empty vector (AdNull), with or without co-treatment with bone morphogenetic protein (BMP)4. Images show cells at time 0 after a vertical scratch has been made and the same area after 96 h of cell migration. N = 3–6 wells per experimental treatment with n = 1–3 areas per well. Average data are shown in Fig 5L. (TIF) [file pone.0165079.s005.tif]

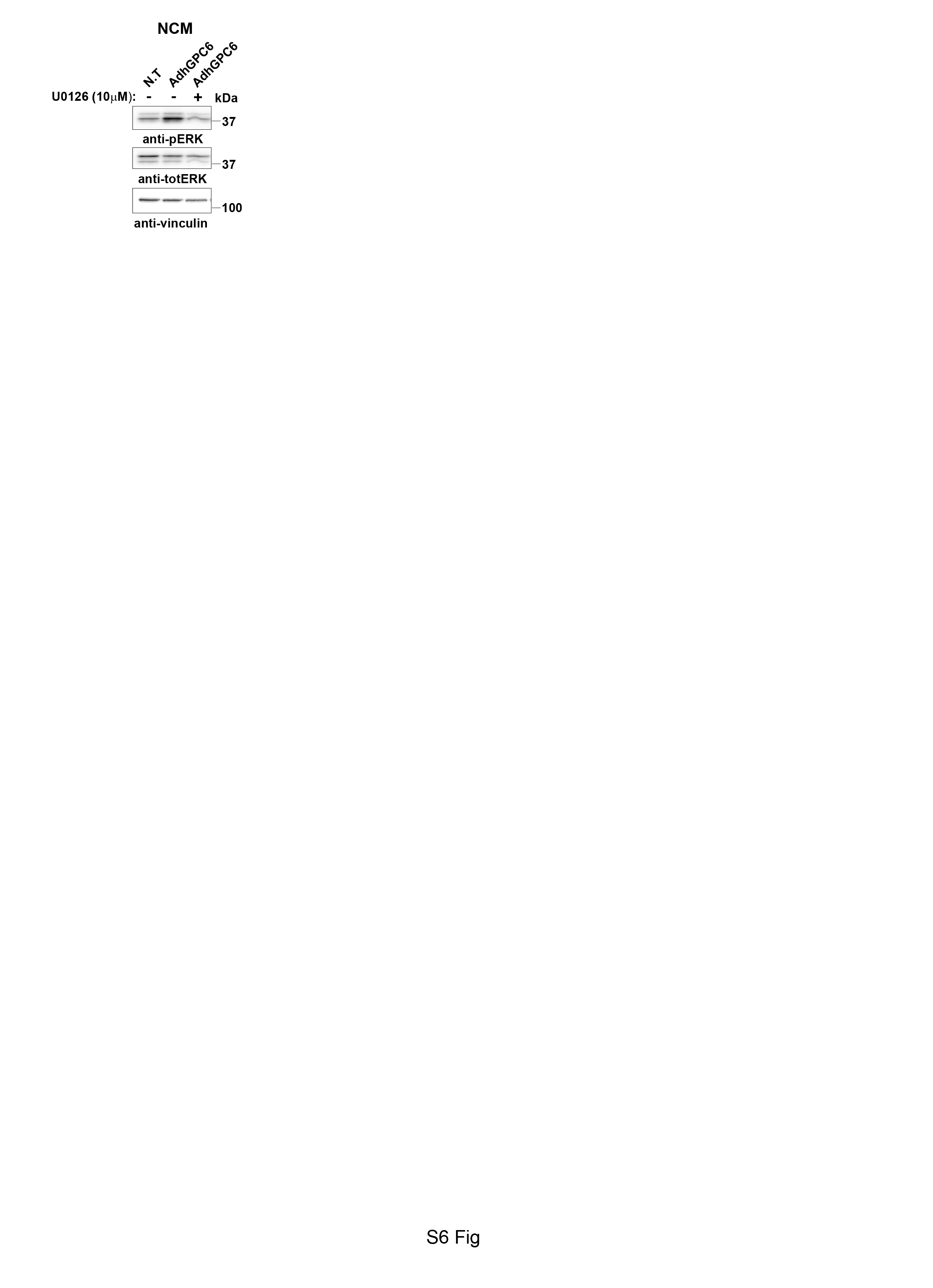

Supplement: S6 Fig — Representative immunoblots of phospho-extracellular signal-regulated kinase (pERK)1 (pERK 44) and total ERK1 (totERK 44; ≈44 kDa), and phospho-ERK2 (pERK 42) and ERK2 (totERK 42; ≈42 kDa) in rat cardiac neonatal cardiomyocytes (NCM) transduced with an adenovirus encoding human GPC6 (AdhGPC6) and in non-transduced controls, analyzed under reducing conditions (+dithiothreitol), n = 3. The dual specificity kinase (MEK1/2) inhibitor U0126 was used to inhibit ERK1/2 activation by phosphorylation. Vinculin was used as loading control. (TIF) [file pone.0165079.s006.tif]
